# Highly efficient 5' capping of mitochondrial RNA with NAD^+^ and NADH by yeast and human mitochondrial RNA polymerase

**DOI:** 10.7554/eLife.42179

**Published:** 2018-12-12

**Authors:** Jeremy G Bird, Urmimala Basu, David Kuster, Aparna Ramachandran, Ewa Grudzien-Nogalska, Atif Towheed, Douglas C Wallace, Megerditch Kiledjian, Dmitry Temiakov, Smita S Patel, Richard H Ebright, Bryce E Nickels

**Affiliations:** 1 Department of Genetics and Waksman InstituteRutgers UniversityUnited States; 2 Department of Chemistry and Waksman InstituteRutgers UniversityUnited States; 3 Department of Biochemistry and Molecular Biology, Robert Wood Johnson Medical SchoolRutgers UniversityUnited States; 4 Biochemistry PhD Program, School of Graduate StudiesRutgers UniversityUnited States; 5 Biochemistry Center HeidelbergHeidelberg UniversityGermany; 6 Department of Cell Biology and NeuroscienceRutgers UniversityUnited States; 7 Center for Mitochondrial and Epigenomic MedicineThe Children’s Hospital of PhiladelphiaUnited States; 8 Department of Pediatrics, Division of Human GeneticsThe Children's Hospital of Philadelphia, Perelman School of MedicineUnited States; 9 Department of Biochemistry and Molecular Biology, Sidney Kimmel Cancer CenterThomas Jefferson UniversityUnited States; Eunice Kennedy Shriver National Institute of Child Health and Human DevelopmentUnited States; Columbia UniversityUnited States

**Keywords:** mitochondria, metabolism, RNA polymerase, transcription initiation, non-canonical initiating nucleotide, RNA capping, *E. coli*, Human, *S. cerevisiae*

## Abstract

Bacterial and eukaryotic nuclear RNA polymerases (RNAPs) cap RNA with the oxidized and reduced forms of the metabolic effector nicotinamide adenine dinucleotide, NAD^+^ and NADH, using NAD^+^ and NADH as non-canonical initiating nucleotides for transcription initiation. Here, we show that mitochondrial RNAPs (mtRNAPs) cap RNA with NAD^+^ and NADH, and do so more efficiently than nuclear RNAPs. Direct quantitation of NAD^+^- and NADH-capped RNA demonstrates remarkably high levels of capping in vivo: up to ~60% NAD^+^ and NADH capping of yeast mitochondrial transcripts, and up to ~15% NAD^+^ capping of human mitochondrial transcripts. The capping efficiency is determined by promoter sequence at, and upstream of, the transcription start site and, in yeast and human cells, by intracellular NAD^+^ and NADH levels. Our findings indicate mtRNAPs serve as both sensors and actuators in coupling cellular metabolism to mitochondrial transcriptional outputs, sensing NAD^+^ and NADH levels and adjusting transcriptional outputs accordingly.

## Introduction

Chemical modifications of the RNA 5'-end provide a layer of ‘epitranscriptomic’ regulation, influencing RNA fate, including stability, processing, localization, and translation efficiency (Höfer and Jäschke, 2018; Jäschke et al., 2016; Ramanathan et al., 2016; Shuman, 2015). One well-characterized RNA 5'-end modification is the ‘cap’ comprising 7-methylguanylate (m^7^G) added to many eukaryotic messenger RNAs (Furuichi and Shatkin, 2000; Shatkin, 1976; Shuman, 1995; Wei et al., 1975). Recently, a new RNA 5'-end cap comprising the metabolic effector nicotinamide adenine dinucleotide (NAD) has been shown to be added to certain RNAs isolated from bacterial, yeast, and human cells (Cahová et al., 2015; Chen et al., 2009; Frindert et al., 2018; Jiao et al., 2017; Walters et al., 2017).

In contrast to a m^7^G cap, which is added to nascent RNA by a capping complex that associates with eukaryotic RNA polymerase II (RNAP II) (Ghosh and Lima, 2010; Martinez-Rucobo et al., 2015; Shuman, 1995; Shuman, 2001; Shuman, 2015), an NAD cap is added by RNAP itself during transcription initiation, by serving as a non-canonical initiating nucleotide (NCIN) (Bird et al., 2016) (reviewed in Barvík et al., 2017; Julius et al., 2018; Vasilyev et al., 2018). NCIN-mediated NAD capping has been demonstrated for bacterial RNAP (Bird et al., 2016; Frindert et al., 2018; Julius and Yuzenkova, 2017; Vvedenskaya et al., 2018) and eukaryotic RNAP II (Bird et al., 2016). Thus, whereas m^7^G capping occurs after transcription initiation, on formation of the ~20th phosphodiester bond, and occurs only in organisms harboring specialized capping complexes, NAD capping occurs in transcription initiation, on formation of the first phosphodiester bond, and because it is performed by RNAP itself, is likely to occur in most, if not all, organisms.

**Figure 1.**
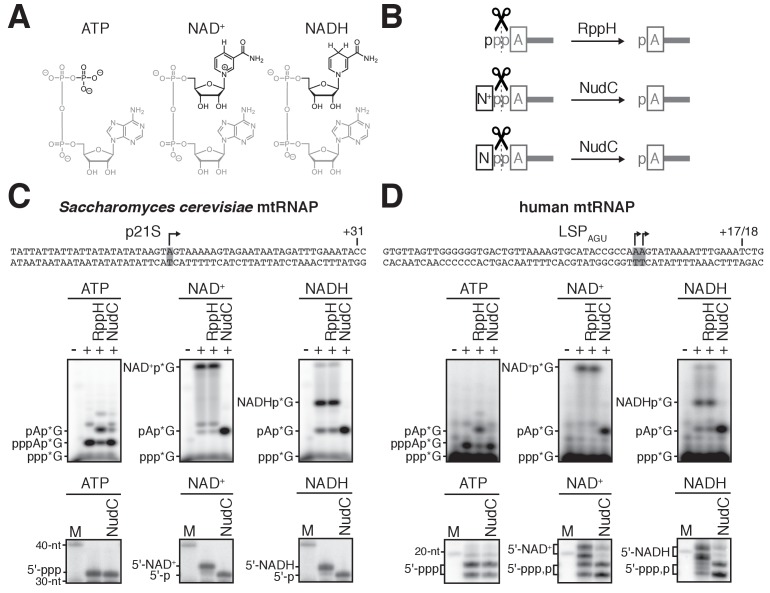
*S. cerevisiae* and human mtRNAPs cap RNA with NAD^+ ^and NADH in vitro. (**A**) Structures of ATP, NAD^+^, and NADH. Grey, identical atoms; black, distinct atoms. (**B**) Processing of RNA 5' ends by RppH and NudC. A, adenosine; N^+^, NAD^+^ nicotinamide; N, NADH nicotinamide; p, phosphate. (**C** and **D**) NCIN capping with NAD^+^ and NADH by *S. cerevisiae* mtRNAP (**C**) and human mtRNAP (**D**). Top, promoter derivatives. Middle, initial RNA products of in vitro transcription reactions with ATP, NAD^+^, or NADH as initiating nucleotide and [α^32^P]-GTP as extending nucleotide. Bottom, full-length RNA products of in vitro transcription reactions with ATP, NAD^+^, or NADH as initiating nucleotide and [α^32^P]-GTP, ATP, UTP, and 3'-deoxy-CTP (**C**), or [α^32^P]-GTP, ATP, and UTP (**D**) as extending nucleotides. Products were treated with RppH or NudC as indicated. Grey box and arrow, transcription start site (TSS);+31 and+17/18, position of last NTP incorporated into full-length RNA products; M, 10-nt marker. 10.7554/eLife.42179.004Figure 1—source data 1.Source data for Figure 1C,D. 10.7554/eLife.42179.005Figure 1—source data 2.Data for Figure 1—figure supplement 1.

NAD exists in oxidized and reduced forms: NAD^+^ and NADH, respectively (Figure 1A). Capping with NAD^+^ has been demonstrated both in vitro and in vivo (Bird et al., 2016; Frindert et al., 2018; Julius and Yuzenkova, 2017; Vvedenskaya et al., 2018). Capping with NADH has been demonstrated in vitro (Bird et al., 2016; Julius and Yuzenkova, 2017).

**Figure 1—figure supplement 1.**
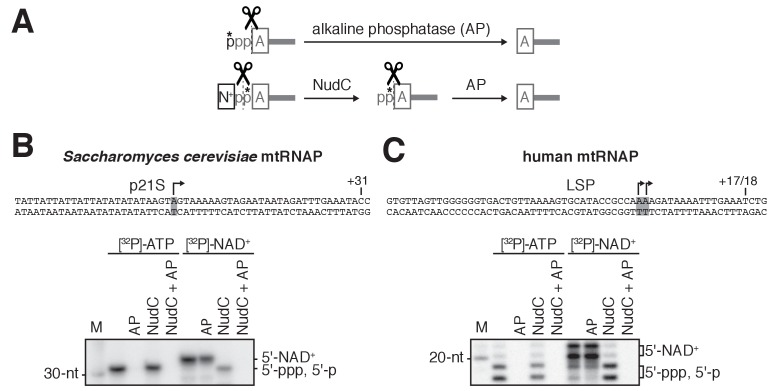
*S. cerevisiae* and human mtRNAPs cap RNA with NAD^+^ *in vitro*: additional data. (**A**) Processing of radiolabeled RNA 5'-ends by alkaline phosphatase (AP) and NudC. A, adenosine; N^+^, NAD^+^ nicotinamide; p, phosphate. *, radiolabeled phosphate. (**B** and **C**). NCIN capping with NAD^+^ by *S. cerevisiae* mtRNAP (**B**) and human mtRNAP (**C**). Top, promoter derivatives. Bottom, full-length RNA products of in vitro transcription reactions with [γ^32^P]-ATP or [α^32^P]-NAD^+^ as initiating nucleotide and GTP, ATP, UTP, and 3'-deoxy-CTP (**C**), or GTP, ATP, and UTP (**D**) as extending nucleotides. Products were treated with NudC alone, AP alone, or NudC and AP, as indicated. Grey box and arrow, TSS; +31 and +17/18, position of last NTP incorporated into RNA; M, 10-nt marker.

Jäschke and co-workers developed a method that combines click-chemistry-mediated covalent capture and high-throughput sequencing, ‘NAD captureSeq,’ to detect NAD^+^-capped RNA (Cahová et al., 2015; Winz et al., 2017). Jäschke and co-workers used this method to identify NAD^+^-capped RNAs in bacterial cells (*Escherichia coli* and *Bacillus subtilis*; Cahová et al., 2015; Frindert et al., 2018). Parker, Kiledjian, and co-workers used the same method to identify NAD^+^-capped RNAs in eukaryotic cells (*Saccharomyces cerevisiae* and human cell line HEK293T; Jiao et al., 2017; Walters et al., 2017). Notably, the identified *Saccharomyces cerevisiae* NAD^+^-capped RNAs included not only RNAs produced by nuclear RNAPs, but also RNAs produced by mitochondrial RNAP (mtRNAP). The eukaryotic nuclear RNAPs--RNAP I, II, and III--are multi-subunit RNAPs closely related in sequence and structure to bacterial RNAP (Cramer, 2002; Darst, 2001; Ebright, 2000; Werner and Grohmann, 2011); in contrast, mtRNAPs are single-subunit RNAPs that are unrelated in sequence and structure to multi-subunit RNAPs and, instead, are related to DNA polymerases, reverse transcriptases, and DNA-dependent RNAPs from T7-like bacteriophages (Cermakian et al., 1996; Cheetham and Steitz, 2000; Hillen et al., 2018; Masters et al., 1987; McAllister and Raskin, 1993; Ringel et al., 2011; Sousa, 1996).

The identification of NAD^+^-capped mitochondrial RNAs in *S. cerevisiae* raises the question of whether eukaryotic single-subunit mtRNAPs--like the structurally unrelated bacterial and eukaryotic nuclear multi-subunit RNAPs--can perform NCIN-mediated capping. A recent review discussed evidence supporting the hypothesis that human mtRNAP can perform NCIN capping (Julius et al., 2018). Here, we show that single-subunit *S. cerevisiae* mtRNAP and human mtRNAP perform NCIN-mediated capping with NAD^+^ and NADH in vitro, and do so substantially more efficiently than bacterial and eukaryotic multi-subunit RNAPs. Further, we show that capping efficiency is determined by promoter sequence, we demonstrate very high levels of NAD^+^ and NADH capping--up to ~60% of mitochondrial transcripts in vivo, and we demonstrate that the extents of capping in vivo, and distributions of NAD^+^ capping vs. NADH capping in vivo are influenced by intracellular levels of NAD^+^ and NADH.

## Results

### *S. cerevisiae* and human mtRNAPs cap RNA with NAD^+^ and NADH in vitro

To assess whether mtRNAP can cap RNA with NAD^+^ and NADH, we performed in vitro transcription experiments (Figure 1 and Figure 1—figure supplement 1). We analyzed *S. cerevisiae* mtRNAP and a DNA template carrying the *S. cerevisiae* mitochondrial 21S promoter (Deshpande and Patel, 2014), and, in parallel, human mtRNAP and a DNA template containing a derivative of the human mitochondrial light-strand promoter, LSP_AGU_ (Sologub et al., 2009) (Figure 1C–D, top). We performed reactions using either ATP, NAD^+^, or NADH as the initiating entity and using [α^32^P]-GTP as the extending nucleotide (Figure 1C–D, middle). We observed efficient formation of an initial RNA product in all cases (Figure 1C–D, middle). The initial RNA products obtained with ATP, but not with NAD^+^ or NADH, were processed by RppH, which previous work has shown to process 5'-triphosphate RNAs to 5'-monophosphate RNAs (Deana et al., 2008) (Figure 1B), whereas the initial RNA products obtained with NAD^+^ or NADH, but not with ATP, were processed by NudC, which previous work has shown to process 5'-NAD^+^- and 5'-NADH-capped RNAs to 5'-monophosphate RNAs (Cahová et al., 2015; Höfer et al., 2016) (Figure 1B). The results establish that *S. cerevisiae* mtRNAP and human mtRNAP are able to generate initial RNA products using NAD^+^ and NADH as NCINs.

We next assessed whether the initial RNA products formed using NAD^+^ and NADH as NCINs can be extended to yield full-length RNA products (Figure 1C–D, bottom, and Figure 1—figure supplement 1). We performed parallel transcription experiments using either ATP, NAD^+^, or NADH as the initiating entity and using [α^32^P]-GTP, ATP, UTP, and 3'-deoxy-CTP (Figure 1C, bottom) or [α^32^P]-GTP, ATP, and UTP (Figure 1D, bottom) as extending nucleotides. We observed efficient formation of full-length RNA products in all cases, and we observed that full-length RNA products obtained with NAD^+^ or NADH, but not with ATP, were sensitive to NudC treatment (Figure 1C–D, bottom). We also observed efficient formation of full-length RNA products in transcription experiments performed using [α^32^P]-ATP or [^32^P]-NAD^+^ as the initiating entity and using non-radiolabeled extending nucleotides (Figure 1—figure supplement 1). Full-length products obtained with [^32^P]-NAD^+^, but not with [α^32^P]-ATP, were insensitive to treatment with alkaline phosphatase (which processes 5' phosphates). Furthermore, NudC treatment of full-length products obtained with [^32^P]-NAD^+^ yielded products that were sensitive to alkaline phosphatase (Figure 1—figure supplement 1). The results establish that *S. cerevisiae* mtRNAP and human mtRNAP not only generate initial RNA products, but also generate full-length RNA products, using NAD^+^ and NADH as NCINs.

### *S. cerevisiae* and human mtRNAPs cap RNA with NAD^+^ and NADH more efficiently than bacterial and nuclear RNAPs

**Figure 2.**
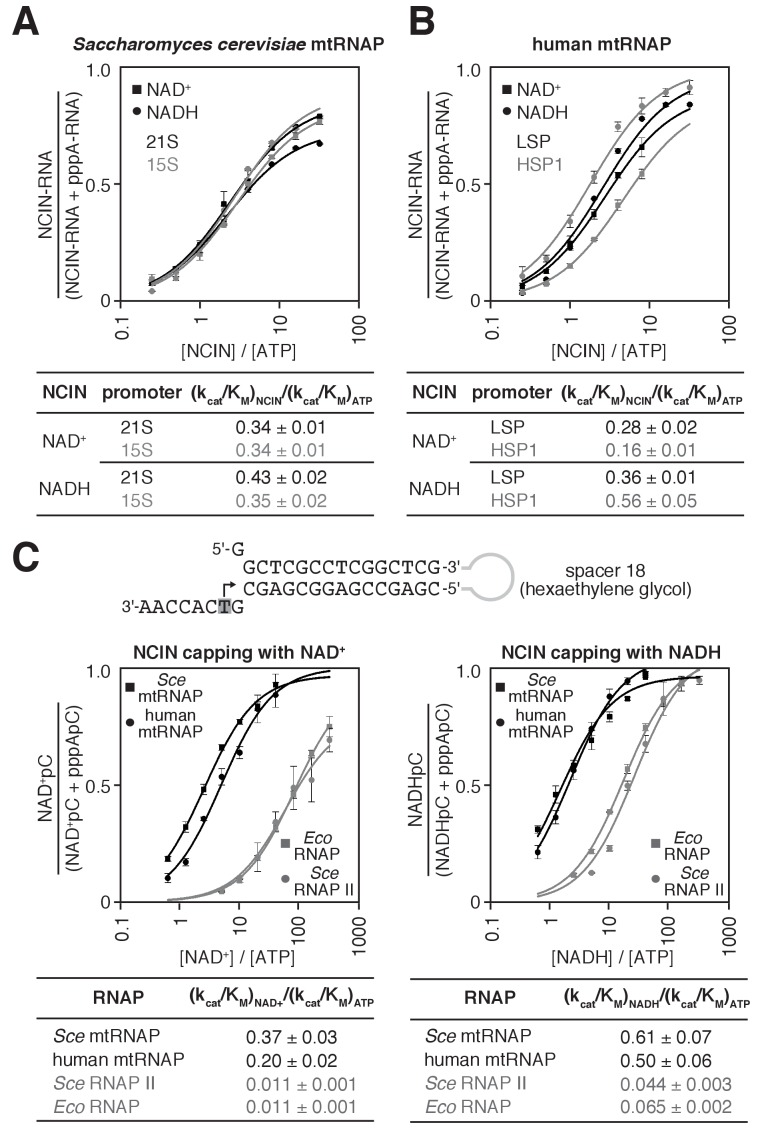
*S. cerevisiae* and human mtRNAPs cap RNA with NAD^+ ^and NADH more efficiently than bacterial and nuclear RNAPs. (**A** and **B**) Dependence of NCIN-mediated capping with NAD^+^ and NADH on [NCIN] / [ATP] ratio for *S. cerevisiae* mtRNAP (**A**) and human mtRNAP (**B**) (mean ± SD; n = 3). DNA templates and representative data are shown in Figure 2—figure supplement 1. (**C**) Dependence of NCIN-mediated capping with NAD^+^ and NADH on [NCIN] / [ATP] ratio for mtRNAPs vs. *E. coli* RNAP and *S. cerevisiae* RNAP II. Top, tailed template. Grey box and arrow indicate TSS. Bottom, dependence of NCIN-mediated capping with NAD^+^ and NADH on [NCIN] / [ATP] ratio for *S. cerevisiae* mtRNAP (*Sce* mtRNAP), human mtRNAP, *E. coli* RNAP (*Eco* RNAP) and *S. cerevisiae* RNAP II (*Sce* RNAP II) (mean ± SD; n = 3). 10.7554/eLife.42179.009Figure 2—source data 1.Data for Figure 2A,B. 10.7554/eLife.42179.010Figure 2—source data 2.Data for Figure 2C,D, and Figure 2—figure supplement 1.

We next determined the relative efficiencies of NCIN-mediated initiation vs. ATP-mediated initiation, (k_cat_/K_M_)_NCIN_ / (k_cat_/K_M_)_ATP_, for mtRNAPs (Figure 2 and Figure 2—figure supplement 1; methods as in Bird et al., 2017). We performed reactions with *S. cerevisiae* mtRNAP and DNA templates carrying the *S. cerevisiae* mitochondrial 21S promoter or 15S promoter (Figure 2A and Figure 2—figure supplement 1), and, in parallel, with human mtRNAP and DNA templates carrying the human mitochondrial light-strand promoter (LSP) or heavy-strand promoter (HSP1) (Figure 2B and Figure 2—figure supplement 1). We obtained values of (k_cat_/K_M_)_NCIN_ / (k_cat_/K_M_)_ATP_ of ~0.3 to ~0.4 for NCIN-mediated initiation with NAD^+^ and NADH by *S. cerevisiae* mtRNAP and ~0.2 to ~0.6 for NCIN-mediated initiation with NAD^+^ and NADH by human mtRNAP. These values imply that NCIN-mediated initiation with NAD^+^ or NADH is up to 40% as efficient as initiation with ATP for *S. cerevisiae* mtRNAP and up to 60% as efficient as initiation with ATP for human mtRNAP.

**Figure 2—figure supplement 1.**
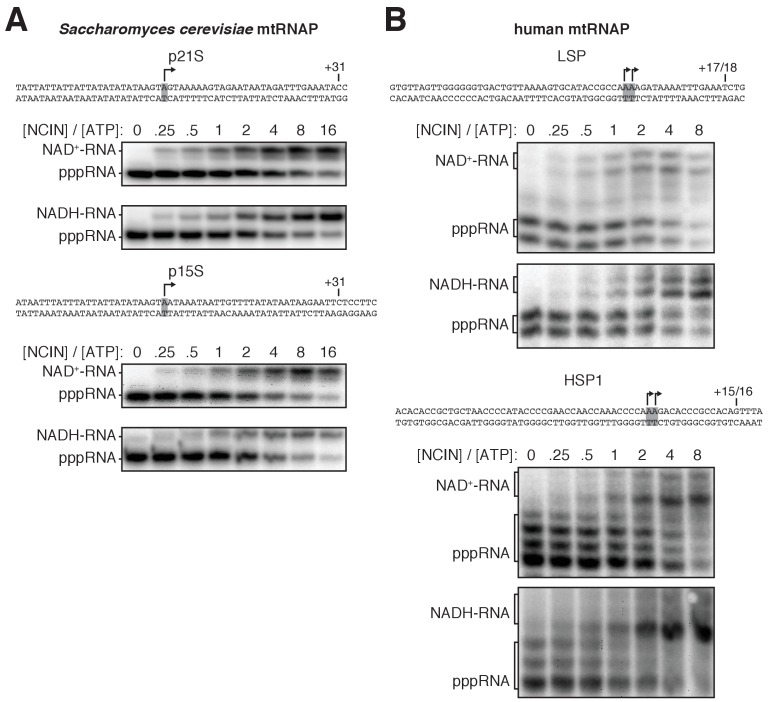
Dependence of NCIN-mediated capping with NAD^+ ^and NADH on [NCIN] / [ATP] ratio for mtRNAPs: representative data. (**A** and **B**) Panels show DNA templates and full-length RNA products of in vitro transcription reactions performed with *S. cerevisiae* mtRNAP (**A**) and human mtRNAP (**B**) with the indicated [NCIN] / [ATP] ratio. Grey box and arrow, TSS; +31, +17/18, +15/16, position of last NTP incorporated into RNA; M, 10-nt marker.

**Figure 2—figure supplement 2.**
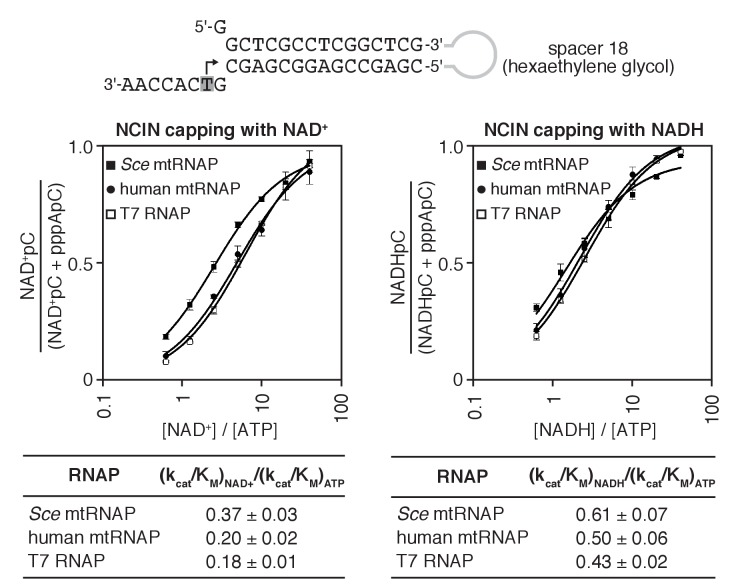
*S. cerevisiae* and human mtRNAPs cap RNA with NAD^+ ^and NADH at least as efficiently as bacteriophage T7 RNAP. Dependence of NCIN-mediated capping with NAD^+^ and NADH on [NCIN] / [ATP] ratio for mtRNAPs vs. T7 RNAP. Top, tailed template. Grey box and arrow indicate TSS. Bottom, Dependence of NCIN-mediated capping with NAD^+^ and NADH on [NCIN] / [ATP] ratio for *S. cerevisiae* mtRNAP (*Sce* mtRNAP), human mtRNAP, and T7 RNAP (mean ± SD; n = 3).

The observed efficiencies of NCIN-mediated initiation with NAD^+^ or NADH by mtRNAPs are substantially higher than the highest previously reported efficiencies for NCIN-mediated initiation with NAD^+^ or NADH by cellular RNAPs (~15%; Bird et al., 2017; Bird et al., 2016; Vvedenskaya et al., 2018). To enable direct comparison of efficiencies of NCIN capping by mtRNAPs vs. cellular RNAPs on the same templates under identical reaction conditions, we performed transcription assays using a ‘tailed’ template (Figure 2C, top) that bypasses the requirement for sequence-specific RNAP-DNA interactions and transcription-initiation factor-DNA interactions for transcription initiation (Dedrick and Chamberlin, 1985; Kadesch and Chamberlin, 1982). In these experiments, we observe efficiencies of NCIN-mediated initiation with NAD^+^ and NADH by mtRNAP that are fully ~10 to ~40 fold higher than efficiencies of NCIN-mediated initiation with NAD^+^ and NADH by *E. coli* RNAP and *S. cerevisiae* RNAP II (Figure 2C, bottom). We conclude that *S. cerevisiae* mtRNAP and human mtRNAP cap RNA with NAD^+^ and NADH more efficiently than bacterial RNAP and eukaryotic nuclear RNAP II.

We next used the same tailed template and reaction conditions as in assays performed with mtRNAPs to determine the efficiency of NCIN-mediated initiation with NAD^+^ and NADH for the single-subunit RNAP of bacteriophage T7 (T7 RNAP) (Figure 2—figure supplement 2). The efficiencies of NCIN-mediated initiation with NAD^+^ and NADH by T7 RNAP were nearly as high as the efficiencies of NCIN-mediated initiation by mtRNAPs. We conclude that there is a quantitative difference in the efficiency of NCIN capping between members of the single-subunit RNAP family (T7 RNAP and mtRNAPs) and members of the multi-subunit RNAP family (bacterial RNAP and eukaryotic nuclear RNAP II).

### Promoter sequence determines efficiency of RNA capping by mtRNAP

In previous work, we have shown that NCIN capping with NAD^+^ and NADH by bacterial RNAP is determined by promoter sequence, particularly at and immediately upstream of, the transcription start site (TSS) (Bird et al., 2016; Vvedenskaya et al., 2018). NCIN capping by bacterial RNAP occurs only at promoters where the base pair (nontemplate-strand base:template-strand base) at the TSS is A:T (+1A promoters), and occurs most efficiently at the subset of +1A promoters where the base pair immediately upstream of the TSS is purine:pyrimidine (−1R promoters). We have further shown that sequence determinants for NCIN capping by bacterial RNAP reside within the template strand of promoter DNA (i.e., the strand that templates incoming nucleotide substrates) (Vvedenskaya et al., 2018).

**Figure 3.**
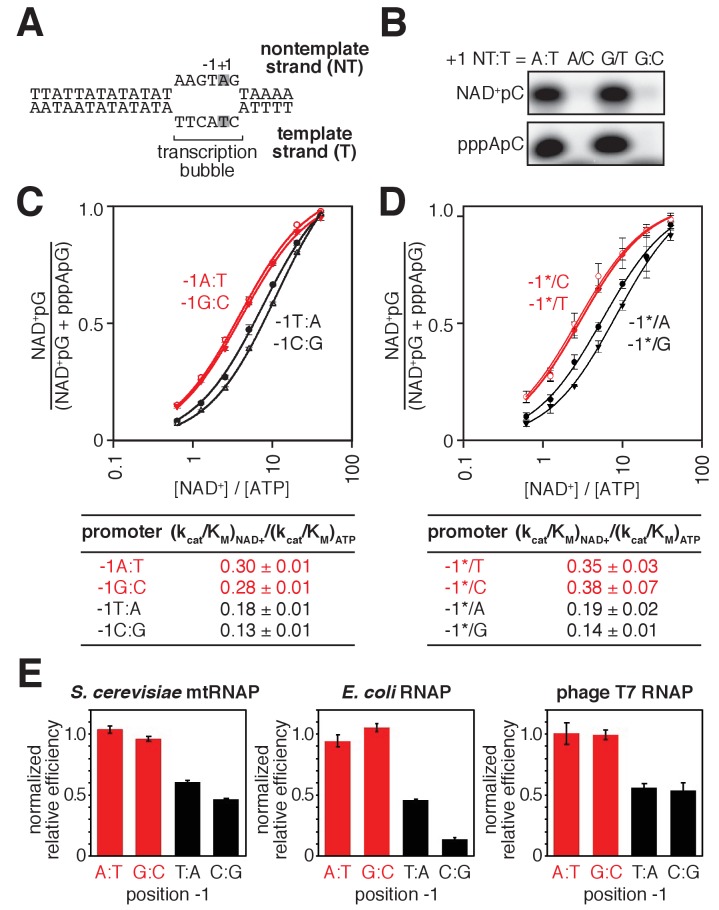
Promoter sequence determines efficiency of RNA capping with NAD^+^ by mtRNAP. (**A**) *S. cerevisiae* mitochondrial 21S promoter DNA depicted in the context of the mtRNAP-promoter open complex. DNA nontemplate strand (NT) on top; DNA template strand (T) on bottom; Unwound, non-base-paired DNA region, ‘transcription bubble,’ indicated by raised and lowered nucleotides; +1 and grey boxes, bases at the TSS; −1, bases immediately upstream of the TSS (the 21S promoter is a −1Y promoter). (**B**) Products of transcription reactions with NAD^+^ as initiating nucleotide and [α^32^P]-CTP as extending nucleotide for templates having complementary or non-complementary nucleotides at position +1. (**C**) Dependence of NAD^+^ capping on [NAD^+^] / [ATP] ratio for homoduplex templates having A:T, G:C, T:A, or C:G at position −1 relative to TSS (mean ± SD; n = 3). Red, −1R promoters; black, −1Y promoters. (**D**) Dependence of NAD^+^ capping on [ATP] / [NAD^+^] ratio for heteroduplex templates having an abasic site (*) on the DNA nontemplate strand (mean ± SD; n = 3). Red, promoters with a template-strand Y; black, promoters with a template-strand R. (**E**) Sequence preferences at position −1 for *S. cerevisiae* mtRNAP, *E. coli* RNAP, and T7 RNAP. Graphs show normalized values of (k_cat_/K_M_)_NAD+_ / (k_cat_/K_M_)_ATP_ determined for homoduplex templates having A:T, G:C, T:A, or C:G at position −1 (mean ± SD; n = 3). Normalized values were calculated by dividing the value for each individual promoter by the average value measured for −1R promoters. Data for *S. cerevisiae* mtRNAP are from panel C, data for *E. coli* RNAP are from (Vvedenskaya et al., 2018), and data for T7 RNAP are from Figure 3—figure supplement 1. 10.7554/eLife.42179.013Figure 3—source data 1.Data for Figure 3B. 10.7554/eLife.42179.014Figure 3—source data 2.Data for Figure 3C,D,E, and Figure 3—figure supplement 1.

To determine whether the specificity for A:T at the TSS (position +1), observed with bacterial RNAP, also is observed with mtRNAP, we assessed NAD^+^ capping by *S. cerevisiae* mtRNAP using promoter derivatives having A:T or G:C at position +1 (Figure 3A–B). We observed NAD^+^ capping in reactions performed using the promoter derivative having A:T at position +1, but not in reactions performed using the promoter derivative having G:C at position +1 (Figure 3B), indicating specificity for A:T at position +1. To determine whether specificity resides in the template strand for A:T at position +1, we analyzed NAD^+^ capping with *S. cerevisiae* mtRNAP using template derivatives having noncomplementary nontemplate- and template-strand-nucleotides (A/C or G/T) at position +1 (Figure 3B). We observed NAD^+^ capping only with the promoter derivative having T as the template strand base at position +1, indicating that specificity at position +1 resides in the template strand.

**Figure 3—figure supplement 1.**
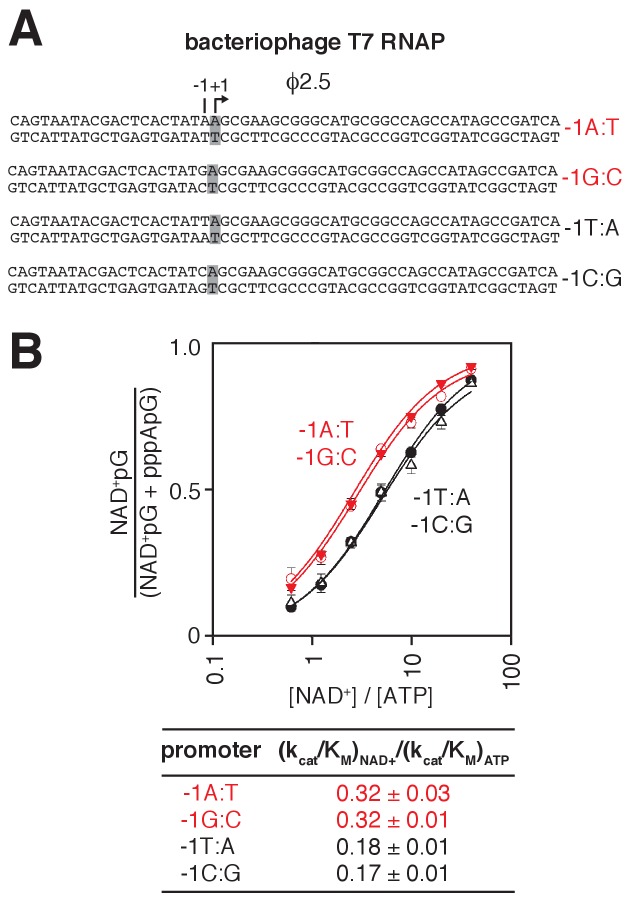
Promoter sequence determines efficiency of RNA capping with NAD^+^: bacteriophage T7 RNAP. (**A**) Bacteriophage T7 RNAP-dependent promoter derivatives analyzed. Red, −1R promoters; black, −1Y promoters; +1 and grey box, bases at the TSS; −1, bases immediately upstream of the TSS. (**B**) Dependence of NAD^+^ capping on [NAD^+^] / [ATP] ratio for homoduplex templates having A:T, G:C, T:A, or C:G at position −1 relative to TSS (mean ± SD; n = 3).

To determine whether specificity for R:Y at position −1, observed with bacterial RNAP, also is observed with mtRNAP, we analyzed NAD^+^ capping by *S. cerevisiae* mtRNAP using promoter derivatives having either R:Y (A:T or G:C) or Y:R (C:G or T:A) at position −1 (Figure 3C). We observed higher efficiencies of NAD^+^ capping with promoter derivatives having R:Y at position −1 than with promoter derivatives having Y:R (Figure 3C). To determine whether specificity at position −1 resides in the DNA template strand, we performed experiments using promoter derivatives having Y (C or T) or R (A or G) at position −1 of the template strand and having an abasic site (*) on the nontemplate strand (Figure 3D). We observed higher efficiencies of NAD^+^ capping in reactions performed using promoter derivatives having Y at template-strand position −1 than with those having R. Furthermore, within error, the capping efficiencies for promoter derivatives having Y or R at template-strand position −1 matched the capping efficiencies for homoduplex promoter derivatives (Figure 3C–D), indicating that sequence information for NAD^+^ capping with *S. cerevisiae* mtRNAP resides exclusively in the template strand.

We conclude that NCIN capping with NAD^+^ by mtRNAP is determined by the sequence at, and immediately upstream of, the TSS (positions +1 and −1, respectively). We further conclude that the sequence and strand preferences at positions +1 and −1 for NCIN capping with NAD^+^ by mtRNAP match the sequence and strand preferences observed for bacterial RNAP (Figure 3C–E) (Bird et al., 2016; Vvedenskaya et al., 2018), suggesting that these sequence and strand preferences may be universal determinants of NCIN capping with NAD^+^ for all RNAPs. Consistent with this hypothesis, we find that sequence preferences for NCIN capping with NAD^+^ by bacteriophage T7 RNAP, another member of the single-subunit RNAP family, match the sequence preferences observed for *S. cerevisiae* mtRNAP and bacterial RNAP (Figure 3E and Figure 3—figure supplement 1). Further consistent with this hypothesis, structural modeling suggests the basis for these sequence and strand preferences is universal: specifically, a strict requirement for template-strand +1T for base pairing to the NAD^+^ adenine moiety, and a preference for template strand −1Y for ‘pseudo’ base pairing to the NAD^+^ nicotinamide moiety (Bird et al., 2016; Vvedenskaya et al., 2018).

### Detection and quantitation of NAD^+^- and NADH-capped mitochondrial RNA in vivo: boronate affinity electrophoresis with processed RNA and synthetic standards

**Figure 4.**
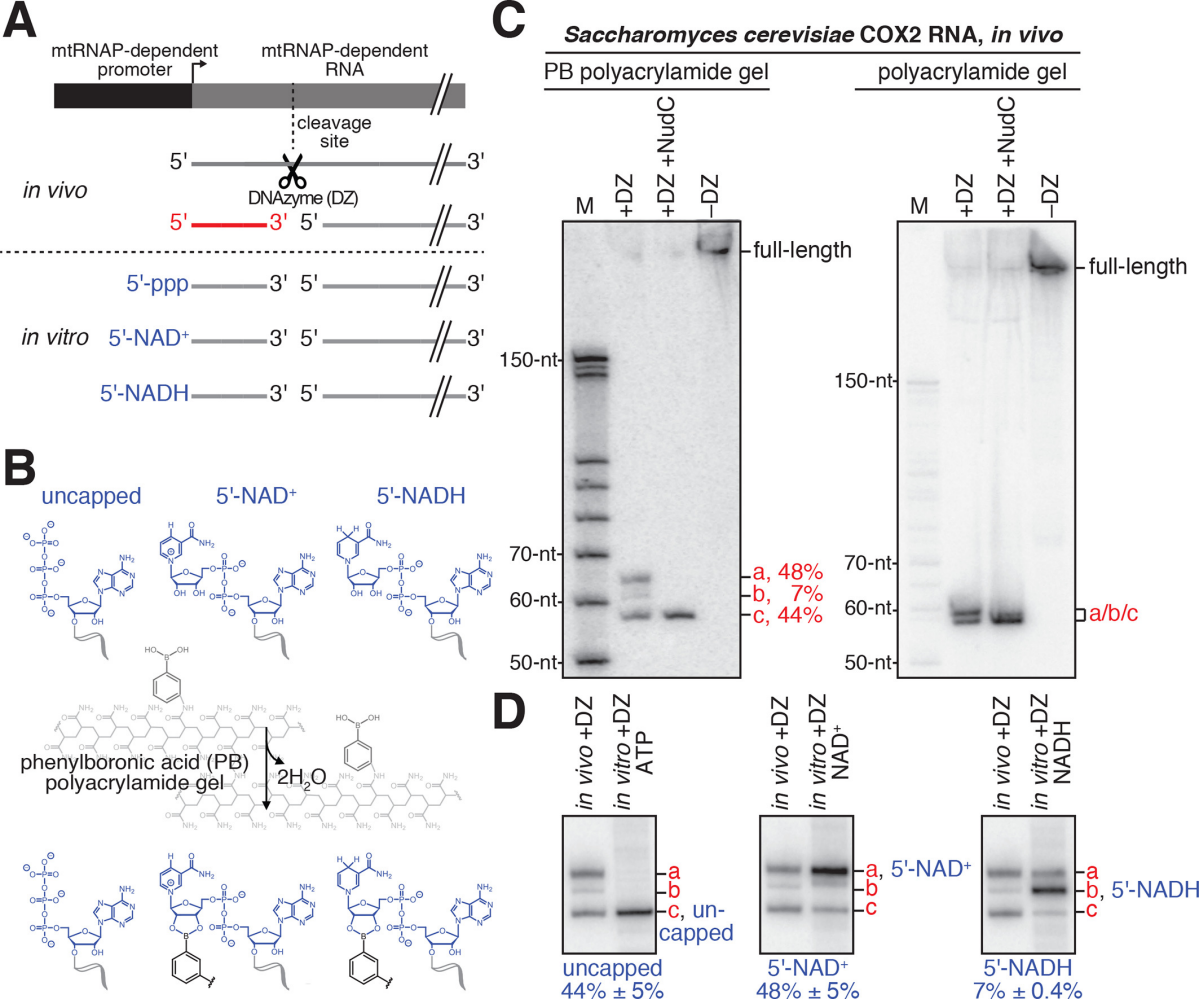
Detection and quantitation of NAD^+^- and NADH-capped mitochondrial RNA in vivo: boronate affinity electrophoresis with DNAzyme-cleaved cellular RNA and DNAzyme-cleaved synthetic NCIN-capped RNA standards. (**A**) Use of DNAzyme (DZ) to process RNA to yield a defined, short 5'-end-containing subfragment, in parallel in vivo (red) and in vitro (blue). Uncapped, 5'-triphosphate (ppp) end generated using ATP-mediated initiation; 5'-NAD^+^, NAD^+^-capped end generated using NAD^+^-mediated initiation; 5'-NADH, NADH-capped end generated using NADH-mediated initiation. (**B**) Use of boronate affinity electrophoresis to resolve 5'-uncapped, 5'-NAD^+^, and 5'-NADH containing RNAs. Grey, structure of phenylboronic acid (PB) polyacrylamide gel. (**C**) PB-polyacrylamide gel (left) and polyacrylamide gel (right) analysis of DNAzyme-generated 5'-end-containing subfragments of *S. cerevisiae* mitochondrial RNA COX2. Red, observed 5'-end-containing RNA subfragments resolved by PB-polyacylamide-gel (left) or not resolved by polyacrylamide gel (right); identities of these subfragments are defined in Panel D. (**D**) Comparison of electrophoretic mobilities of observed 5'-end-containing subfragments of COX2 RNA generated in vivo to 5'-end-containing subfragments of synthetic RNA standards generated in vitro. a, NAD^+^-capped RNA; b, NADH-capped RNA; c, uncapped RNA (mean ± SD; n = 3). 10.7554/eLife.42179.016Figure 4—source data 1.Data for Figure 4D.

Kössel, Jäschke and co-workers have demonstrated that boronate affinity electrophoresis allows resolution of uncapped RNAs from capped RNAs--such as m^7^G, NAD^+^ and NADH--that contain a vicinal-diol moiety (Igloi and Kössel, 1985; Igloi and Kössel, 1987; Nübel et al., 2017). However, the procedures of Kössel, Jäschke and co-workers have two limitations: (i) boronate affinity electrophoresis does not allow resolution of RNAs longer than ~200 nt, and (ii) boronate affinity electrophoresis, by itself, is unable to distinguish between different vicinal-diol containing cap structures (m^7^G, NAD^+^, NADH, or others). Here, to overcome these limitations, we have combined boronate affinity electrophoresis with use of oligodeoxynucleotide-mediated RNA cleavage (‘DNAzyme’ cleavage) (Joyce, 2001) and use of synthetic NCIN-capped RNA standards generated using NCIN-mediated transcription initiation in vitro (Figure 4). Use of DNAzyme cleavage enables processing of long RNAs to yield defined, short, 5'-end-containing subfragments (Figure 4A). Use of synthetic NCIN-capped RNA standards enables distinction between capped species (Figure 4A,D).

To detect and quantify NCIN capping with NAD^+^ and NADH in mitochondrial RNA isolated from cells, we employed the following steps: (i) DNAzyme cleavage of target RNAs to generate 5'-end-containing subfragments < 80 nt in length (Figure 4A, top); (ii) DNAzyme treatment of synthetic NAD^+^- and NADH-capped RNA standards having sequences identical to RNAs of interest (Figure 4A, bottom); (iii) boronate affinity electrophoresis of DNAzyme-generated subfragments of mitochondrial RNA and DNAzyme-generated subfragments of synthetic NAD^+^- and NADH-capped RNA standards having sequences identical to RNAs of interest (Figure 4B); and (iv) detection of DNAzyme-generated 5'-end-containing subfragments of mitochondrial RNAs and synthetic RNA standards by hybridization with a radiolabeled oligodeoxyribonucleotide probe (Figure 4C–D).

**Figure 5.**
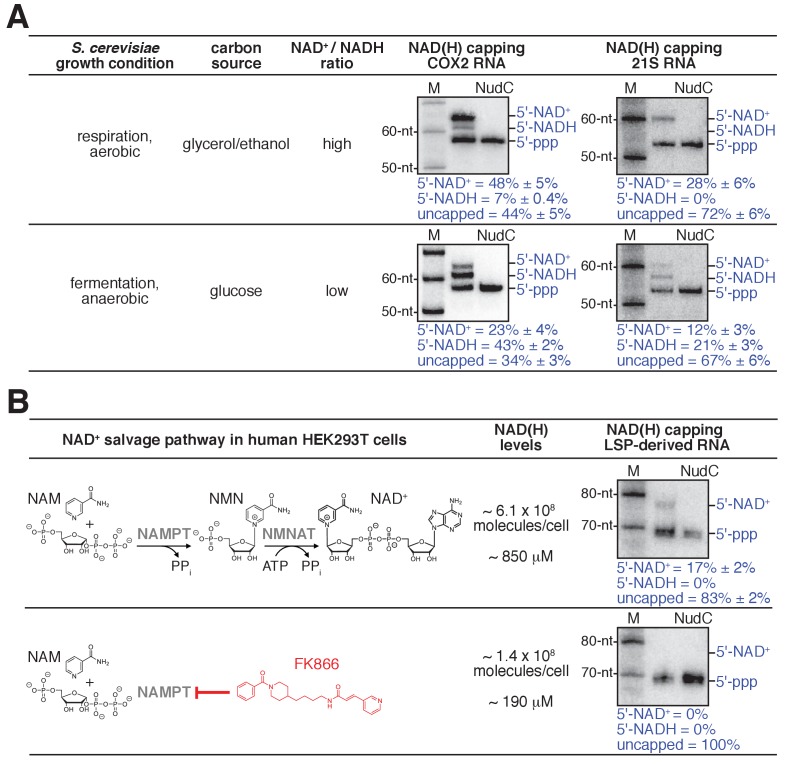
Detection and quantitation of NAD^+^- and NADH-capped mitochondrial RNA in vivo: effects of intracellular NAD^+ ^and NADH levels in *S. cerevisiae* and human cells. (**A**) Changes in intracellular NAD^+^/NADH ratios result in changes in levels of NAD^+^- and NADH-capped mitochondrial RNA (mean ± SD; n = 3). Gel images show representative data for *S. cerevisiae* COX2 RNA (left) and 21S RNA (right). Blue annotations as in Figure 4. (**B**) Changes in intracellular NAD(H) levels result in changes in levels of NAD^+^- and NADH-capped mitochondrial RNA (mean ± SD; n = 3). Gel images show representative data for LSP-derived RNAs. Red, NAD(H) biosynthesis inhibitor FK866; NAMPT, Nicotinamide phosphoribosyltransferase; NMNAT, Nicotinamide mononucleotide adenylyltransferase. 10.7554/eLife.42179.018Figure 5—source data 1.Data for Figure 5 (gels). 10.7554/eLife.42179.019Figure 5—source data 2.Data for Figure 5 (values of NCIN capping).

We selected for analysis two *S. cerevisiae* mitochondrial RNAs that previously had been detected as NAD^+^-capped: COX2 and 21S (Walters et al., 2017). We isolated *S. cerevisiae* total RNA and analyzed COX2 and 21S RNAs using the procedure described in the preceding paragraph. The results are presented in Figures 4C–D and 5A (top). For both COX2 and 21S RNAs, we detect at least one RNA species with electrophoretic mobility retarded as compared to that of uncapped RNA, indicating the presence of capped RNA. Treatment with the decapping enzyme NudC eliminates these species, confirming the presence of capping. Comparison of the electrophoretic mobilities to those of synthetic NAD^+^- and NADH-capped RNA standards indicates that one of the capped species is NAD^+^-capped RNA, and the other capped species, present under these growth conditions only for COX2 RNA, is NADH-capped RNA. The results show that both COX2 and 21S RNAs are present in NAD^+^-capped forms, and that COX2 RNA also is present in an NADH-capped-form. Because the hybridization probe detects RNA fragments that contain 5' ends generated by transcription initiation (red subfragment depicted in Figure 4A), the detected NAD^+^ and NADH caps are concluded to be at 5' ends generated by transcription initiation, as opposed to 5' ends generated by RNA processing. The results confirm that *S. cerevisiae* mitochondrial RNAs undergo NAD^+^ capping in cells, show that *S. cerevisiae* mitochondrial RNAs undergo NAD^+^ capping at 5' ends generated by transcription initiation (as opposed to 5' ends generated by RNA processing), and show that *S. cerevisiae* mitochondrial RNAs also undergo NADH capping in cells.

The observed levels of NAD^+^- and NADH-capping of *S. cerevisiae* mitochondrial RNAs are remarkably high. For COX2 RNA, NAD^+^-capped RNA comprises ~50% of the total COX2 RNA pool and NADH-capped RNA comprises ~10% of the total COX2 RNA pool (Figures 4C–D and 5A, top). For 21S RNA, NAD^+^-capped RNA comprises ~30% of the total 21S RNA pool (Figure 5A, top). These levels of NCIN capping are ~2 to ~50 times higher than levels of NCIN-capping in exponentially growing *E. coli* (less than 1% to ~20% for NAD^+^-capping; not previously detected for NADH-capping) (Bird et al., 2016; Cahová et al., 2015; Nübel et al., 2017; Vvedenskaya et al., 2018).

We performed analogous experiments analyzing RNAs produced by transcription from the human mitochondrial LSP promoter (Figure 5B, top). We isolated and analyzed total RNA from HEK293T cells. We observed an NAD^+^-capped species comprising ~15% of the total LSP-derived RNA pool (Figure 5B, top). The results establish that human mitochondrial RNAs undergo NAD^+^ capping in cells and show that human mitochondrial RNAs undergo NAD^+^ capping at 5' ends generated by transcription initiation (as opposed to 5' ends generated by RNA processing).

### Detection and quantitation of NAD^+^- and NADH-capped mitochondrial RNA in vivo: mtRNAPs serve as both sensors and actuators in coupling cellular metabolism to mitochondrial gene expression

Mitochondria are the primary locus of metabolism and energy transformation in the eukaryotic cell, serving as the venue for the tricarboxylic acid cycle (TCA) cycle and oxidative phosphorylation. The TCA cycle reduces NAD^+^ to NADH and oxidative phosphorylation oxidizes NADH to NAD^+^. Our finding that mtRNAPs perform NCIN capping with NAD^+^ and NADH at efficiencies that vary in a simple mass-action-dependent fashion with [NAD^+^] / [ATP] and [NADH] / [ATP] ratios in vitro (Figures 1 and 2), and our finding that mtRNAPs perform efficient NCIN capping to yield NAD^+^- and NADH-capped mitochondrial RNAs in vivo (Figures 4 and 5), raise the possibility that mtRNAPs may serve as both sensors and actuators in coupling metabolism to mitochondrial gene expression in vivo.

As a first test of this hypothesis, we assessed whether changing intracellular [NAD^+^] / [NADH] ratios results in changes in NAD^+^ and NADH capping of mitochondrial RNAs. We isolated total RNA from *S. cerevisiae* grown under conditions that result in either high or low [NAD^+^] / [NADH] ratios (Bekers et al., 2015; Canelas et al., 2008): respiration (glycerol/ethanol; aerobic) or fermentation (glucose; anaerobic). We analyzed the same two mitochondrial RNAs as above: COX2 and 21S (Figure 5A). We observed marked changes in levels of NAD^+^ and NADH capping for both analyzed mitochondrial RNAs. For COX2 RNA, on changing from the growth condition yielding a high [NAD^+^] / [NADH] ratio to the growth condition yielding a low [NAD^+^] / [NADH] ratio, we observe a decrease in levels of NAD^+^ capping (from ~50% to ~20%) and an anti-correlated increase in the level of NADH capping (from ~10% to ~40%). Notably, the total level of NAD^+^ and NADH capping, NAD(H) capping, remains constant under the two conditions (~60%), indicating that the relative levels of NCIN-mediated initiation and ATP-mediated initiation do not change (Figure 5A). For 21S RNA, the same pattern is observed (Figure 5A): on changing from the growth condition yielding a high [NAD^+^] / [NADH] ratio to the growth condition yielding a low [NAD^+^] / [NADH] ratio, the level of NAD^+^ capping decreases (from ~30% to ~10%), the level of NADH capping increases (from 0% to ~20%), and the total level of NAD^+^ and NADH capping remains constant (~30%). The results indicate that changing the [NAD^+^] / [NADH] ratio changes transcription outputs in vivo.

As a second test of this hypothesis, we assessed whether changing intracellular total NAD(H) levels results in changes in NCIN capping of mitochondrial RNAs (Figure 5B). We isolated RNA from human HEK293T cells grown under conditions yielding either high or low intracellular NAD(H) levels: standard growth media or growth media in the presence of the NAD(H)-biosynthesis inhibitor FK866 (Hasmann and Schemainda, 2003; Khan et al., 2006). We analyzed the same LSP-derived mitochondrial RNA as in the preceding section (Figure 5B). On changing from the growth condition yielding high intracellular NAD(H) levels to the growth condition yielding low NAD(H) levels, we observe a marked change in the total level of NCIN capping (from ~15% to 0%). The results indicate that changing NAD(H) levels changes levels of NCIN-capped mitochondrial RNAs in vivo.

Taken together, the results of the two experiments in Figure 5 indicate that mtRNAP serves as both sensor and actuator in coupling [NAD^+^] / [NADH] ratios to relative levels of NAD^+^- and NADH-capped mitochondrial RNAs (Figure 5A), and in coupling total NAD(H) levels to total levels of NCIN-capped mitochondrial RNAs (Figure 5B), thereby coupling cellular metabolism to mitochondrial transcription outputs. We suggest that mtRNAPs serve as sensors through their mass-action-dependence in selecting NAD^+^ vs. NADH vs. ATP as the initiating entity during transcription initiation, and serve as actuators by incorporating NAD^+^ vs. NADH vs. ATP at the RNA 5' end during transcription initiation.

## Discussion

Our results show that *S. cerevisiae* and human mtRNAPs cap RNA with NAD^+^ and NADH (Figure 1), show that *S. cerevisiae* and human mtRNAPs cap RNA with NAD^+^ and NADH more efficiently than bacterial and eukaryotic nuclear RNAPs (Figure 2), and show that capping efficiency by mtRNAPs is determined by promoter sequence (Figure 3). Our results further show that the proportions of mitochondrial RNAs that are capped with NAD^+^ and NADH are remarkably high--up to ~60% (Figures 4 and 5)--and that these proportions change in response to cellular NAD^+^ and NADH levels (Figure 5).

We and others previously have shown that NCIN capping by cellular RNAPs has functional consequences, including modulating RNA stability and modulating RNA translatability (Bird et al., 2016; Cahová et al., 2015; Jiao et al., 2017). Our results here showing that *S. cerevisiae* and human mitochondrial RNAs are capped at substantially higher levels than non-mitochondrial RNAs--up to ~60% for analyzed *S. cerevisiae* mitochondrial RNAs and up to ~15% for analyzed human mitochondrial RNAs (Figures 4 and 5)--suggest that NCIN capping in mitochondria occurs at a higher efficiency, and has a higher importance, than NCIN capping in other cellular compartments. Four other considerations support this hypothesis. First, mtRNAPs are substantially more efficient at NAD^+^ and NADH capping than bacterial and eukaryotic nuclear RNAPs (Figure 2C). Second, levels of NAD^+^ and NADH relative to ATP in mitochondria are substantially higher than levels of NAD^+^ and NADH relative to ATP in bacteria and eukaryotic nuclei (Cambronne et al., 2016; Chen et al., 2016; Park et al., 2016). Third, all *S. cerevisiae* and human mitochondrial promoters are +1A promoters (18 promoters in *S. cerevisiae* mitochondria; two promoters in human mitochondria) (Biswas, 1999; Chang and Clayton, 1984; Taanman, 1999), in contrast to bacterial and eukaryotic nuclear RNAP promoters, for which approximately half are +1A promoters (Chen et al., 2013; Haberle et al., 2014; Saito et al., 2013; Thomason et al., 2015; Tsuchihara et al., 2009). Fourth, we observe capping with both NAD^+^ and NADH for mitochondrial RNAs in vivo (Figures 4 and 5), whereas, to date, capping has been observed with only NAD^+^ for non-mitochondrial RNAs in vivo, raising the possibility that, in mitochondria, but not in other cellular compartments, NAD^+^ and NADH caps dictate different RNA fates and, correspondingly, different transcription outputs.

Our results showing that levels of NAD^+^ and NADH capping by mtRNAP correlate with changes in intracellular levels of NAD^+^ and NADH (Figure 5) indicate that mtRNAP serves as a sensor, sensing [NAD^+^] / [ATP] and [NADH] / [ATP] ratios, when selecting initiating entities and, simultaneously, serves as an actuator by altering RNA 5' ends when selecting initiating entities. Because all *S. cerevisiae* and human mitochondrial promoters are +1A promoters, this dual sensor/actuator activity of mtRNAP will occur at, and couple metabolism to gene expression at, all *S. cerevisiae* and human mitochondrial promoters. This dual sensor/actuator activity of mtRNAP obviates the need for a dedicated signal-processing machinery for coupling metabolism to gene expression, instead employing a pan-eukaryotic housekeeping enzyme for signal processing. As such, this dual sensor/actuator activity of mtRNAP provides a remarkably economical, parsimonious mechanism of coupling metabolism to gene expression.

## Materials and methods

**Key resources table:** 

Reagent type (species) or resource	Designation	Source or reference	Identifiers	Additional information
Strain, strain background (*E. coli*)	BL21(DE3) bacteria	NEB	C2527H	
Strain, strain background (*E. coli*)	NiCo21(DE3) bacteria	NEB	C2529H	
Strain, strain background (*E. coli*)	Artic Express (DE3) bacteria	Fisher Scientific	NC9444283	
Strain, strain background (*S. cerevisiae*)	246.1.1 (*MATa ura3 trp1 leu2 his4*)	Gift of A. Vershon		
Cell line (human)	HEK293T (human embryonic kidney cells)	ATCC	CRL-3216	
Recombinant DNA reagent	pIA900	Gift of I. Artsimovitch		
Recombinant DNA reagent	pET NudC-His	(Bird et al., 2016)		
Recombinant DNA reagent	pJJ1399	gift of J. Jaehning		
Recombinant DNA reagent	pTrcHisC-Mtf1	gift of J. Jaehning		
Recombinant DNA reagent	pPROEXHTb-POLRMT (43–1230)−6xHis	(Ramachandran et al., 2017)		
Recombinant DNA reagent	pPROEXHTb-TFAM (43-245)−6xHis	(Ramachandran et al., 2017)		
Recombinant DNA reagent	pT7TEV-HMBP4	(Yakubovskaya et al., 2014)		
Recombinant DNA reagent	pAR1219	(Jia et al., 1996)		
Sequence-based reagent	DK64	Integrated DNA Technologies (IDT)	tailed template with PEG_6_ linker	GGCTCGCCTCGGCTCG/iSp18/ CGAGCCGAGGCGAGCGTCACCAA
Sequence-based reagent	JB459	IDT	human LSP DNA template + 1 AGU variant nontemplate strand	GTGTTAGTTGGGGGGTGACTGTT AAAAGTGCATACCGCCAAAGTATA AAATTTGTGGGCC
Sequence-based reagent	JB460	IDT	human LSP DNA template + 1 AGU variant template strand	GGCCCACAAATTTTATACTTTGGC GGTATGCACTTTTAACAGTCACCC CCCAACTAACAC
Sequence-based reagent	JB469	IDT	T7φ2.5–35 n nontemplate strand (−1T)	CAGTAATACGACTCACTATTAGCGAA GCGGGCATGCGGCCAGCCATAGC CGATCA
Sequence-based reagent	JB470	IDT	T7φ2.5–35 n template strand (−1A)	TGATCGGCTATGGCTGGCCGCATGCC CGCTTCGCTAATAGTGAGTCGTA TTACTG
Sequence-based reagent	JB471	IDT	T7φ2.5–35 n nontemplate strand (−1A)	CAGTAATACGACTCACTATAAGCGAAGC GGGCATGCGGCCAGCCATAG CCGATCA
Sequence-based reagent	JB472	IDT	T7φ2.5–35 n template strand (−1T)	TGATCGGCTATGGCTGGCCGCATGCCC GCTTCGCTTATAGTGAGTCGTATTACTG
Sequence-based reagent	JB473	IDT	T7φ2.5–35 n nontemplate strand (−1G)	CAGTAATACGACTCACTATGAGCGAAG CGGGCATGCGGCCAGCCATAG CCGATCA
Sequence based reagent	JB474	IDT	T7φ2.5 35 n template strand (−1C)	TGATCGGCTATGGCTGGCCGCATGCC CGCTTCGCTCATAGTGAGTCGTATTACTG
Sequence-based reagent	JB475	IDT	T7φ2.5–35 n nontemplate strand (−1C)	CAGTAATACGACTCACTATCAGCGAA GCGGGCATGCGGCCAGCCA TAGCCGATCA
Sequence-based reagent	JB476	IDT	T7φ2.5–35 n template strand (−1G)	TGATCGGCTATGGCTGGCCGCATGC CCGCTTCGCTGATAGTG AGTCGTATTACTG
Sequence-based reagent	JB515	IDT	probe for human LSP-generated RNA (complementary to positions + 2 to+31)	CACCAGCCTAACCAGATTTCAA ATTTTATC
Sequence-based reagent	JB525	IDT	probe for *S. cerevisiae* 21S RNA (complementary to positions + 9 to+42)	CTATATAATAAATATTTCAAATC TATTATTCTAC
Sequence-based reagent	JB526	IDT	*S. cerevisiae* 21S RNA DNAzyme; cleaves transcript at position + 53	ACTCCATGATTAGGCTAGCTACAA CGACTCTTTAAATCT
Sequence-based reagent	JB555	IDT	probe for *S. cerevisiae* COX2 RNA (complementary to positions + 8 to+46)	ATCTTAACCTTTAGACTCTTTTGTC TATTTATAATATGT
Sequence-based reagent	JB557	IDT	*S. cerevisiae* COX2 DNAzyme; cleaves at position + 57	TCTTAATAAATCTAAGGCTAGCTACA ACGAATTTTAATAAATCTT
Sequence-based reagent	JB559	IDT	human LSP-generated RNA DNAzyme; cleaves at position + 67	GCACTTAAACAGGCTAGCTACAA CGAATCTCTGCCA
Sequence-based reagent	JB560	IDT	*S. cerevisiae* COX2 −40 to + 125 nontemplate strand oligo (for generation of in vitro transcription template)	TATATAATAATAAATTATAAATAAATTTT AATTAAAAGTAGTATTAACATATTATAAA TAGACAAAAGAGTCTAAAGGTTAAGATT TATTAAAATGTTAGATTTATTAAGATTAC AATTAACAAC
Sequence-based reagent	JB561	IDT	*S. cerevisiae* COX2 −40 to + 3 forward primer (for generation of in vitro transcription template)	TATATAATAATAAATTATAAATAAATTTT AATTAAAAGTAGT
Sequence-based reagent	JB562	IDT	*S. cerevisiae* COX2 + 83 to+125 reverse primer (for generation of in vitro transcription template)	GTTGTTAATTGTAATCTTAATAAATCTAA CATTTTAATAAATC
Sequence-based reagent	UB1	IDT	human LSP DNA template (−43 to + 19) nontemplate strand	ATGTGTTAGTTGGGGGGTGACTGTTAA AAGTGCATACCGCCAAAAGATAAAATT TGAAATCTG
Sequence-based reagent	UB2	IDT	human LSP DNA template (−43 to + 19) template strand	CAGATTTCAAATTTTATCTTTTGGCGGT ATGCACTTTTAACAGTCACCCCCCAAC TAACACAT
Sequence-based reagent	UB3	IDT	human HSP1 DNA template (−43 to + 20) nontemplate strand	ACACACCGCTGCTAACCCCATACCCCGA ACCAACCAAACCCCAAAGACACCCGCC ACAGTTTA
Sequence-based reagent	UB4	IDT	human HSP1 DNA template (−43 to + 20) template strand	TAAACTGTGGCGGGTGTCTTTGGGGT TTGGTTGGTTCGGGGTATGGGGTTA GCAGCGGTGTGT
Sequence-based reagent	UB5	IDT	*S. cerevisiae* 15S DNA template (−25 to + 1; C-less cassette) nontemplate strand	ATAATTTATTTATTATTATATAAGTAAT AAATAATTGTTTTATATAATAAGAA TTCTCCTTC
Sequence-based reagent	UB6	IDT	*S. cerevisiae* 15S DNA template (−25 to + 1; C-less cassette) template strand	GAAGGAGAATTCTTATTATATAAAACA ATTATTTATTACTTATATAATAATAA ATAAATTAT
Sequence-based reagent	UB7	IDT	*S. cerevisiae* 21S DNA template (−25 to + 1; C-less cassette) nontemplate strand	TATTATTATTATTATATATATAAGTAG TAAAAAGTAGAATAATAGATTT GAAATACC
Sequence-based reagent	UB8	IDT	*S. cerevisiae* 21S DNA template (−25 to + 1; C-less cassette) template strand	GAAGGAGACCAACCACAAACACACA ACAACCACCAACTACTTATATAATAA TAAATAAATTAT
Sequence-based reagent	UB9	IDT	*S. cerevisiae* 21S DNA template (−25 to + 1; C-less and A-less cassette) nontemplate strand	ATAATTTATTTATTATTATATAAGTAG TTGGTGGTTGTTGTGTGTTTGTG GTTGGTCTCCTTC
Sequence-based reagent	UB10	IDT	*S. cerevisiae* 21S DNA template (−25to + 1; C-less and A-less cassette) template strand	GAAGGAGACCAACCACAAACACAC AACAACCACCAACTACTTATATAA TAATAAATAAATTAT
Sequence-based reagent	UB11	IDT	*S. cerevisiae* 21S nontemplate strand (−1A)	ATAATTTATTTATTATTATATAAGAA GTTGGTGGTTGTTGTGTGTTTGTG GTTGGTCTCCTTC
Sequence-based reagent	UB12	IDT	*S. cerevisiae* 21S template strand (−1T)	GAAGGAGACCAACCACAAACACA CAACAACCACCAACTTCTTATATA ATAATAAATAAATTAT
Sequence-based reagent	UB13	IDT	*S. cerevisiae* 21S nontemplate strand (−1G)	ATAATTTATTTATTATTATATAAGG AGTTGGTGGTTGTTGTGTGTTTG TGGTTGGTCTCCTTC
Sequence-based reagent	UB14	IDT	*S. cerevisiae* 21S template strand (−1C)	GAAGGAGACCAACCACAAACACA CAACAACCACCAACTCCTTATAT AATAATAAATAAATTAT
Sequence-based reagent	UB15	IDT	*S. cerevisiae* 21S nontemplate strand (−1C)	ATAATTTATTTATTATTATATAAG CAGTTGGTGGTTGTTGTGTGT TTGTGGTTGGTCTCCTTC
Sequence-based reagent	UB16	IDT	*S. cerevisiae* 21S template strand (−1G)	GAAGGAGACCAACCACAAACACA CAACAACCACCAACTGCTTATATA ATAATAAATAAATTAT
Sequence-based reagent	UB17	IDT	*S. cerevisiae* 21S nontemplate strand (+1C)	ATAATTTATTTATTATTATATAAGTC GTTGGTGGTTGTTGTGTGTTTGT GGTTGGTCTCCTTC
Sequence-based reagent	UB18	IDT	*S. cerevisiae* 21S template strand (+1G)	GAAGGAGACCAACCACAAACACAC AACAACCACCAACGACTTATATAA TAATAAATAAATTAT
Sequence-based reagent	JB527	IDT	*S. cerevisiae* 21S nontemplate strand (−1 abasic)	ATAATTTATTTATTATTATATAAG/ idSp/AGTTGGTGGTTGTTGTGTGT TTGTGGTTGGTCTCCTTC
Peptide, recombinant protein (*S. cerevisiae*)	Rpo41 (mtRNAP)	(Tang et al., 2009)		
Peptide, recombinant protein (*S. cerevisiae*)	Mtf1	(Paratkar and Patel, 2010)		
Peptide, recombinant protein (Human)	POLRMT (mtRNAP)	(Ramachandran et al., 2017)		
Peptide, recombinant protein (Human)	TFAM	(Ramachandran et al., 2017)		
Peptide, recombinant protein (Human)	TFB2	(Yakubovskaya et al., 2014)		
Peptide, recombinant protein (*S. cerevisiae*)	RNA polymerase II	Gift of C. Kaplan		
Peptide, recombinant protein (*E. coli*)	RNA polymerase core (β'−6xHis)	(Artsimovitch et al., 2003)		
Peptide, recombinant protein	T7 RNA polymerase	(Jia et al., 1996)		
Peptide, recombinant protein (*E. coli*)	NudC	(Cahová et al., 2015)		
Peptide, recombinant protein	Phusion Flash HF master mix	ThermoFisher	F-548L	
Peptide, recombinant protein	T4 Polynucleotide Kinase	NEB	M0201L	
Peptide, recombinant protein	RNA 5' pyrophosphohydrolase (RppH)	NEB	M0356S	
Peptide, recombinant protein	FastAP Alkaline Phosphatase	Thermo Fisher	EF0651	
Commercial assay or kit	Monarch PCR and DNA clean up kit	NEB	T1030S	
Chemical compound, drug	Nuclease-free water (not DEPC-treated)	ThermoFisher	AM9932	
Chemical compound, drug	Bacto agar	VWR	90000–760	
Chemical compound, drug	Bacto tryptone	VWR	90000–286	
Chemical compound, drug	Bacto yeast extract	VWR	90000–726	
Chemical compound, drug	D-Glucose monhydrate	Amresco	0643–1 kg	
Chemical compound, drug	Glycerol	EMD Millipore	55069521	
Chemical compound, drug	DMEM medium	Thermo Fisher	11965–092	
Chemical compound, drug	Fetal Bovine Serum	Atlanta Biological	S11150H	
Chemical compound, drug	dNTP solution mix, 10 mM of each NTP	NEB	N0447S	
Chemical compound, drug	NTP set (ultra-pure), 100 mM solutions	GE Healthcare	27-2025-01	
Chemical compound, drug	NAD^+^	Roche (Sigma-Aldrich)	10127965001	
Chemical compound, drug	NADH	Roche (Sigma-Aldrich)	10107735001	
Chemical compound, drug	Tris base (Amresco)	VWR	97061–800	
Chemical compound, drug	Boric Acid (ACS grade)	VWR	97061–980	
Chemical compound, drug	EDTA disodium salt dyhydrate	VWR	97061–018	
Chemical compound, drug	0.5 M EDTA pH 8	ThermoFisher	AM9260G	
Chemical compound, drug	Dibasic Sodium phosphate	EMD Millipore	SX0715-1	
Chemical compound, drug	Sodium Chloride	EMD Millipore	SX0420-3	
Chemical compound, drug	Potassium Chloride	EMD Millipore	7300–500 GM	
Chemical compound, drug	Sodium Citrate	EMD Millipore	7810–1 KG	
Chemical compound, drug	Sodium Acetate, trihydrate	VWR	MK736406	
Chemical compound, drug	Ficoll 400	VWR	AAB22095-18	
Chemical compound, drug	Polyvinylpyrrolidone	EMD Millipore	7220–1 KG	
Chemical compound, drug	Diethyl Pyrocarbonate (DEPC)	VWR	AAB22753-14	
Chemical compound, drug	Formamide, deionized	VWR	EM-4610	
Chemical compound, drug	Sodium dodecylsulfate (SDS)	VWR	97064–470	
Chemical compound, drug	Magnesium chloride hexahydrate	VWR	EM-5980	
Chemical compound, drug	Magnesium sulfate heptahydrate	VWR	EM-MX0070-1	
Chemical compound, drug	Glycerol (ACS grade)	VWR	EMGX0185-5	
Chemical compound, drug	Bovine Serum Albumin (BSA) fraction V	VWR	101174–932	
Chemical compound, drug	Bromophenol Blue	VWR	EM-BX1410-7	
Chemical compound, drug	Xylene Cyanol	Sigma-Aldrich	X4126-10G	
Chemical compound, drug	Amaranth Dye	VWR	200030–400	
Chemical compound, drug	Temed (JT Baker)	VWR	JT4098-1	
Chemical compound, drug	Ammonium Persulfate	VWR	97064–594	
Chemical compound, drug	Dithiothreitol (DTT)	Gold Bio	DTT50	
Chemical compound, drug	Glycogen from Oyster (type II)	Sigma-Aldrich	G8751	
Chemical compound, drug	Hydrochloric Acid (ACS plus)	Fisher Scientific	A144-212	
Chemical compound, drug	Ethyl Alcohol	Pharmco-AAPER	111000200	
Chemical compound, drug	GeneMate LE Quick Dissolve agarose	BioExpress	E-3119–500	
Chemical compound, drug	SequaGel sequencing system	National Diagnostics	EC833	
Chemical compound, drug	Nytran SuPerCharge Nylon Membrane	VWR	10416296	
Chemical compound, drug	SigmaSpin G25 cleanup columns	Sigma-Aldrich	S5059	
Chemical compound, drug	^32^P NAD^+^ 250 uCi	Perkin Elmer	BLU023X250UC	
Chemical compound, drug	γ-^32^P ATP Easy Tide 1 mCi	Perkin Elmer	BLU502Z001MC	
Chemical compound, drug	α-^32^P CTP Easy Tide 250 uCi	Perkin Elmer	BLU508H250UC	
Chemical compound, drug	α-^32^P GTP Easy Tide 250 uCi	Perkin Elmer	BLU506H250UC	
Chemical compound, drug	α-^32^P UTP Easy Tide 250 uCi	Perkin Elmer	BLU507H250UC	
Chemical compound, drug	Decade Marker	Thermo Fisher	AM7778	
Chemical compound, drug	TRI Reagent	Molecular Research Center	TR118	
Chemical compound, drug	Acid phenol:chloroform (CHCl_3_) pH 4.5	ThermoFisher	AM9720	
Chemical compound, drug	FK866 hydrochloride hyrate	Sigma-Aldrich	F8557	
Software, algorithm	Excel	Microsoft	365	
Software, algorithm	ImageQuant	GE Healthcare	TL 5.1, TL v8.2	
Software, algorithm	SigmaPlot	Systat Software Inc.	Version 10	
Software, algorithm	Pymol	Schrodinger, LLC	http://www.pymol.org	
Software, algorithm	Illustrator	Adobe	Version CS6	
Other	Typhoon RBG Imager	GE Healthcare		
Other	NanoDrop 2000C spectrophotometer	Thermo Fisher		
Other	UV Crosslinker	Fisher Scientific	FB-UVXL-1000	
Other	Hybridization oven 5420	VWR	97005–252	
Other	Sequi-Gen GT sequencing systems (21 × 50) (38 × 30)	Bio-Rad	1653871 and 1653873	

### Proteins

*S. cerevisiae* mtRNAP (Rpo41) was prepared from *E. coli* strain BL21(DE3) transformed with pJJ1399 (gift of Judith A. Jaehning) using culture and induction procedures, polyethyleneimine treatment, ammonium sulfate precipitation, Ni-sepharose, DEAE sepharose and Heparin-sepharose chromatography as in (Tang et al., 2009). *S. cerevisiae* Mtf1 was prepared from *E. coli* strain BL21(DE3) transformed with pTrcHisC-Mtf1 (Paratkar and Patel, 2010) and purified using culture and induction procedures, polyethyleneimine treatment, ammonium sulfate precipitation, and tandem DEAE sepharose and Ni-sepharose chromatography as in (Tang et al., 2009).

Human mtRNAP (POLRMT) and TFAM were purified from *E. coli* strain BL21(DE3) transformed with pPROEXHTb-POLRMT(43–1230)−6xHis (Ramachandran et al., 2017) or pPROEXHTb-TFAM(43-245)−6xHis (Ramachandran et al., 2017), respectively, using culture and induction procedures, polyethyleneimine treatment, ammonium sulfate precipitation, and Ni-sepharose and heparin-sepharose chromatography as in (Ramachandran et al., 2017). Human TFB2 was purified from *E. coli* strain ArcticExpress (DE3) (Stratagene) transformed with pT7TEV-HMBP4 (Yakubovskaya et al., 2014), using culture and induction procedures, polyethyleneimine treatment, ammonium sulfate precipitation, Ni-sepharose and heparin-sepharose chromatography, and size exclusion chromatography as in (Yakubovskaya et al., 2014).

T7 RNAP was prepared from *E. coli* strain BL21 transformed with pAR1219 using culture and inductions procedures, SP-Sephadex, CM-Sephadex and DEAE-Sephacel chromatography as described in (Jia et al., 1996).

*E. coli* RNAP core enzyme was prepared from *E. coli* strain NiCo21(DE3) transformed with plasmid pIA900 (Artsimovitch et al., 2003) using culture and induction procedures, immobilized-metal-ion affinity chromatography on Ni-NTA agarose, and affinity chromatography on Heparin HP as in (Artsimovitch et al., 2003).

*S. cerevisiae* RNA polymerase II core enzyme (gift of Craig Kaplan) was prepared as described in (Barnes et al., 2015).

*E. coli* NudC was prepared from *E. coli* strain NiCo21(DE3) transformed with plasmid pET NudC-His (Bird et al., 2016) using metal-ion chromatography and size-exclusion chromatography as in (Cahová et al., 2015).

RNA 5' pyrophosphohydrolase (RppH) and T4 polynucleotide kinase (PNK) were purchased from New England Biolabs (NEB). FastAP Thermosensitive Alkaline Phosphatase was purchased from Thermo Fisher Scientific. Molar concentrations of purified proteins were determined by light absorbance at 280 nm and the calculated respective molar extinction coefficients.

### Oligodeoxyribonucleotides

Sequences of the oligodeoxyribonucleotides used in this work are provided in Key Resources Table. All oligodeoxyribonucleotides were purchased from Integrated DNA Technologies (IDT) with standard desalting purification unless otherwise specified.

Linear in vitro transcription templates used for transcription assays shown in Figures 1, 2 and 3 were generated by mixing complementary equimolar amounts of nontemplate- and template-strand DNA in 10 mM Tris HCl pH 8.0, incubating the mixture at 95°C for 5 min, and cooling the mixture by 0.5°C per minute to 25°C.

Transcription templates used to generate in vitro RNA standards for Northern analysis (Figures 4 and 5) were generated by PCR. Reactions contained a mixture of 5 nM template oligo, 0.5 µM forward primer, 0.5 µM reverse primer, and Phusion HF Master Mix (Thermo Scientific). Reaction products were isolated using a Monarch PCR and DNA cleanup kit (NEB).

The radiolabeled 10-nt marker labeled ‘M’ in gel images shown in Figures 1, 4 and 5 was generated using the Decade Marker System (Thermo Fisher Scientific), PNK (NEB) and [γ^32^P]-ATP (Perkin Elmer; 6,000 Ci/mmol).

### In vitro transcription assays

Assays performed with *S. cerevisiae* mtRNAP were based on procedures described in (Deshpande and Patel, 2014). Assays performed with human mtRNAP were based on procedures described in (Ramachandran et al., 2017).

For initial RNA product assays in Figure 1C, Figure 1—figure supplement 1, and Figure 3, 1 µM DNA template, 1 µM *S. cerevisiae* mtRNAP, and 1 µM Mtf1 were incubated at 25°C for 10 min in *Sce*-mtRNAP reaction buffer (50 mM Tris-acetate pH 7.5, 100 mM potassium glutamate, 10 mM magnesium acetate, 0.01% Tween-20, 1 mM DTT, and 5% glycerol). A mixture containing the initiating nucleotide (200 µM ATP, 1 mM NAD^+^, or 1 mM NADH) and extending nucleotide (10 µM of non-radiolabeled GTP plus 6 mCi of [α^32^P]-GTP [Perkin Elmer; 3,000 Ci/mmol]) was added, and assays were incubated at 25°C for 30 min. For assays in Figure 1C, Figure 1—figure supplement 1 and radiolabeled initial products were isolated using a Nanosep 3 kDa cutoff spin concentrator (Pall). For assays in Figure 3B, reactions were stopped with 10 µl RNA loading dye (95% deionized formamide, 18 mM EDTA, 0.25% SDS, xylene cyanol, bromophenol blue, amaranth) and were analyzed by electrophoresis on 7.5 M urea, 1x TBE, 20% polyacrylamide gels (UreaGel System; National Diagnostics), followed by storage-phosphor imaging (Typhoon 9400 variable-mode imager; GE Life Science).

For the initial RNA product assays in Figure 1D and Figure 1—figure supplement 1, 1 µM DNA template, 1 µM human mtRNAP, 1 µM TFAM, and 1 µM TFB2M were incubated at 25°C for 10 min in human-mtRNAP reaction buffer (50 mM Tris-acetate pH 7.5, 50 mM sodium glutamate, 10 mM magnesium acetate, 1 mM DTT, and 0.05% Tween-20). A mixture containing the initiating nucleotide (200 µM ATP, 1 mM NAD^+^, or 1 mM NADH) and extending nucleotide (10 µM of non-radiolabeled GTP plus 6 mCi of [α^32^P]-GTP at [Perkin Elmer; 3,000 Ci/mmol]) was added, and assays were incubated at 25°C for 60 min. Radiolabeled initial RNA products were isolated using a Nanosep 3 kDa cutoff spin concentrator (Pall).

A portion of the recovered initial RNA products were mixed with either 10 U of RppH or 400 nM NudC and incubated at 37°C for 30 min. Reactions were stopped by addition of 10 µl RNA loading dye. Samples were analyzed by electrophoresis on 7.5 M urea, 1x TBE, 20% polyacrylamide gels (UreaGel System; National Diagnostics), followed by storage-phosphor imaging (Typhoon 9400 variable-mode imager; GE Life Science).

For full-length product assays in Figure 1C and Figure 1—figure supplement 1 (panel B), 1 µM DNA template, 1 µM *S. cerevisiae* mtRNAP, and 1 µM Mtf1 were incubated at 25°C for 10 min in *Sce*-mtRNAP reaction buffer. A mixture containing the initiating nucleotide (1 mM ATP, 1 mM NAD^+^, or 1 mM NADH for Figure 1C; 200 µM non-radiolabeled ATP plus 10 µCi [γ^32^P]-ATP [Perkin Elmer; 6,000 Ci/mmol] or 1 mM NAD^+^ plus 20 µCi [α^32^P]-NAD^+^ [Perkin Elmer; 800 Ci/mmol] for Figure 1—figure supplement 1) and extending nucleotides (200 µM GTP, 200 µM 3'-deoxy-CTP, 20 µM ATP, 200 µM non-radiolabeled UTP, and 6 mCi of [α^32^P]-UTP [Perkin Elmer; 3000 Ci/mmol] for Figure 1C; 200 µM GTP, 200 µM 3'-deoxy-CTP, 20 µM ATP, 200 µM UTP for Figure 1—figure supplement 1) was added, and assays were incubated at 25°C for 30 min. Reactions were stopped by addition of stop solution (0.6 M Tris HCl pH 8.0, 18 mM EDTA, 0.1 mg/ml glycogen), samples were extracted with acid phenol:chloroform (5:1, pH 4.5; Thermo Fisher Scientific), and RNA products were recovered by ethanol precipitation and resuspended in NudC reaction buffer (10 mM Tris HCl pH 8.0, 50 mM NaCl, 10 mM MgCl_2_, 1 mM DTT).

For full-length product assays in Figure 1D and Figure 1—figure supplement 1 (panel C), 1 µM DNA template, 1 µM human mtRNAP, 1 µM TFAM, and 1 µM TFB2M were incubated at 25°C for 10 min in human-mtRNAP reaction buffer. A mixture containing the initiating nucleotide (1 mM ATP, 1 mM NAD^+^, or 1 mM NADH for Figure 1D; 200 µM non-radiolabeled ATP plus 10 µCi [γ^32^P]-ATP [Perkin Elmer; 6000 Ci/mmol] or 1 mM NAD^+^ plus 20 µCi [α^32^P]-NAD^+^ [Perkin Elmer; 800 Ci/mmol] for Figure 1—figure supplement 1) and extending nucleotides (200 µM GTP, 20 µM ATP, 200 µM non-radiolabeled UTP, and 6 mCi of [α^32^P]-UTP [Perkin Elmer; 3000 Ci/mmol] for Figure 1D; 200 µM GTP, 20 µM ATP, 200 µM UTP for Figure 1—figure supplement 1) was added, and assays were incubated at 25°C for 60 min. Reactions were stopped by addition of stop solution, samples were extracted with acid phenol:chloroform (5:1) (pH 4.5; Thermo Fisher Scientific), RNA products were recovered by ethanol precipitation and resuspended in NudC reaction buffer.

Full-length RNA products were incubated at 37°C for 30 min with 400 nM NudC alone (Figure 1C–D and Figure 1—figure supplement 1), 0.25 U FastAP Thermosensitive Alkaline Phosphatase alone (Figure 1—figure supplement 1), or both NudC and FastAP (Figure 1—figure supplement 1). Reactions were stopped by addition of an equal volume of RNA loading dye. Samples were analyzed by electrophoresis on 7.5 M urea, 1x TBE, 20% polyacrylamide gels (UreaGel System; National Diagnostics), followed by storage-phosphor imaging (Typhoon 9400 variable-mode imager; GE Life Science).

### Determination of efficiency of NCIN-mediated initiation vs. ATP-mediated initiation, (k_cat_/K_M_)_NCIN_ / (k_cat_/K_M_)_ATP,_in vitro: full-length product assays

For experiments in Figure 2A–B and Figure 2—figure supplement 1, 1 µM of template DNA, 1 µM of mtRNAP, and 1 µM transcription factor(s) (Mtf1 for *S. cerevisiae* mtRNAP; TFAM and TFB2M for human mtRNAP) were incubated at 25°C for 10 min in *Sce*-mtRNAP or human reaction buffer. A mixture containing 200 µM ATP, 200 µM UTP, 200 µM non-radiolabeled GTP, and 6 mCi [α^32^P]-GTP at 3000 Ci/mmol and NCIN (0, 50, 100, 200, 400, 800, 1600, 3200, 6400 µM) was added, and assays were incubated at 25°C for 30 min. Reactions were stopped by addition of an equal volume of RNA loading dye. Samples were analyzed by electrophoresis on 7.5 M urea, 1x TBE, 20% polyacrylamide gels (UreaGel System; National Diagnostics) supplemented with 0.2% 3-acrylamidophenylboronic acid (Boron Molecular), followed by storage-phosphor imaging (Typhoon 9400 variable-mode imager; GE Life Science).

Bands corresponding to uncapped (pppRNA) and NCIN-capped (NCIN-RNA) full-length products were quantified using ImageQuant software. The ratio of NCIN-RNA to total RNA [NCIN-RNA / (pppRNA + NCIN RNA)] was plotted vs. the relative concentrations of NCIN vs. ATP ([NCIN] / [ATP]) on a semi-log plot (SigmaPlot) and non-linear regression was used to fit the data to the equation: y = (ax) / (b + x); where y is [NCIN-RNA / (pppRNA + NCIN RNA)], x is ([NCIN] / [ATP]), and a and b are regression parameters. The resulting fit yields the value of x for which y = 0.5. The relative efficiency (k_cat_/K_M_)_NCIN_ / (k_cat_/K_M_)_ATP_ is equal to 1/x. Data for determination of relative efficiencies are means of three technical replicates.

### Determination of efficiency of NCIN-mediated initiation vs. ATP-mediated initiation, (k_cat_/K_M_)_NCIN_ / (k_cat_/K_M_)_ATP,_in vitro: initial product assays (Bird et al., 2017)

For experiments in Figure 3C–D, and Figure 3—figure supplement 1, 1 µM of template DNA, 1 µM of RNAP, and 1 µM transcription factor(s) (Mtf1 for *S. cerevisiae* mtRNAP; TFAM and TFB2M for human mtRNAP; none for T7 RNAP) were incubated at 25°C for 10 min in *Sce*-mtRNAP, human mtRNAP reaction buffer, or T7 RNAP reaction buffer (40 mM Tris HCl pH 7.9, 6 mM MgCl_2_, 2 mM DTT, 2 mM Spermidine). A mixture containing 1 mM NCIN, ATP (0, 25, 50, 100, 200, 400, 800, 1600 µM), 20 µM non-radiolabeled GTP, and 6 mCi [α^32^P]-GTP at 3000 Ci/mmol was added, and assays were incubated at 25°C for 30 min. Reactions were stopped by addition of an equal volume of RNA loading dye. Samples were analyzed by electrophoresis on 7.5 M urea, 1x TBE, 20% polyacrylamide gels (UreaGel System; National Diagnostics) supplemented with 0.2% 3-acrylamidophenylboronic acid (Boron Molecular), followed by storage-phosphor imaging (Typhoon 9400 variable-mode imager; GE Life Science).

For experiments in Figure 2C and Figure 2—figure supplements 2, 200 nM of tailed template and 500 nM RNAP (*S. cerevisiae* mtRNAP, human mtRNAP, *S. cerevisiae* RNAP II, T7 RNAP, or *E. coli* RNAP) were incubated at 25°C for 15 min in reaction buffer containing 10 mM Tris pH 8.0, 50 mM potassium glutamate, 10 mM MgCl_2_, 2 mM DTT, and 50 ug/ml BSA. A mixture containing NCIN (1 mM NCIN for mtRNAPs, T7 RNAP, and *E. coli* RNAP; 4 mM for *S. cerevisiae* RNAP II), ATP (0, 25, 50, 100, 200, 400, 800, 1600 µM for mtRNAPs and *S. cerevisiae* RNAP II; 0, 6.25, 12.5, 25, 50, 100, 200, 400 µM for *E. coli* RNAP), 10 µM non-radiolabeled CTP, and 6 mCi [α^32^P]-CTP (Perkin Elmer; 3000 Ci/mmol) was added, and assays were incubated at 25°C for 1 hr. Reactions were stopped by addition of an equal volume of RNA loading dye. Samples were analyzed by electrophoresis on 7.5 M urea, 1x TBE, 20% polyacrylamide gels (UreaGel System; National Diagnostics), followed by storage-phosphor imaging (Typhoon 9400 variable-mode imager; GE Life Science).

Bands corresponding to uncapped (pppApC) and NCIN-capped (NCINpC) initial RNA products were quantified using ImageQuant software. The ratio of NCINpC to total RNA (NCINpC / [pppApC + NCINpC]) was plotted vs. the relative concentrations of NCIN vs. ATP ([NCIN] / [ATP]) on a semi-log plot (SigmaPlot) and non-linear regression was used to fit the data to the equation: y = (ax) / (b + x); where y is [NCINpC / (pppApC + NCINpC)], x is ([NCIN] / [ATP]), and a and b are regression parameters. The resulting fit yields the value of x for which y = 0.5. The relative efficiency (k_cat_/K_M_)_NCIN_ / (k_cat_/K_M_)_ATP_ is equal to 1/x. Data for determination of relative efficiencies are means of three technical replicates.

### Detection and quantitation of NAD^+^- and NADH-capped mitochondrial RNA in vivo: isolation of total cellular RNA from *S. cerevisiae*

For analysis of NAD^+^ and NADH capping during respiration, *S. cerevisiae* strain 246.1.1 [(Tatchell et al., 1981); *MAT*α *ura3 trp1 leu2 his4*; gift of Andrew Vershon, Rutgers University] was grown at 30°C in 25 ml YPEG (24 g Bacto-tryptone, 20 g Bacto-yeast extract, 30 mL ethanol, 3% glycerol per liter) in 125 ml flasks (Bellco) shaken at 220 rpm. When cell density reached an OD600 ~1.8 (approximately 24 hr) the cell suspension was centrifuged to collect cells (5 min, 10,000 g at 4°C), supernatants were removed, and cell pellets were resuspended in 0.8 mL RNA extraction buffer (0.5 mM NaOAc pH 5.5, 10 mM EDTA, 0.5% SDS).

For analysis of NAD^+^ and NADH capping during fermentation, *S. cerevisiae* strain 246.1.1 was grown at 30°C in 100 ml YPD [24 g Bacto-tryptone, 20 g Bacto-yeast extract, 2% (w/v) glucose per liter] in 125 ml flasks (Bellco) with airlocks to prevent oxygenation without shaking for 42 hr. The cell suspension was centrifuged to collect cells (5 min, 10,000 g at 4°C), supernatants were removed, and cell pellets were resuspended in 0.8 mL RNA extraction buffer (0.5 mM NaOAc pH 5.5, 10 mM EDTA, 0.5% SDS).

To extract RNA, an equal volume of acid phenol:chloroform (5:1, pH 4.5; Thermo Fisher Scientific) was added to cells in resuspension buffer and mixed by vortexing. Samples were incubated at 65°C for 5 min, −80°C for 5 min, then centrifuged (15 min, 21,000 g, 4°C) to separate the aqueous and organic phases. The aqueous phase was collected and acid phenol:chloroform extraction was performed two more times on this solution. RNA transcripts were recovered by ethanol precipitation and resuspended in RNase free H_2_O.

### Detection and quantitation of NAD^+^- and NADH-capped mitochondrial RNA in vivo: isolation of total cellular RNA from human cells

Human embryonic kidney HEK293T cells (obtained from ATCC, tested negative for mycoplasma) were maintained under 5% CO_2_ at 37°C in DMEM medium (Thermo Fisher Scientific) supplemented with 10% fetal bovine serum (Atlanta Biologicals), 100 units/ml penicillin, and 100 µg/ml streptomycin. HEK293T cells were seeded in 100 mm tissue-culture treated plates and grown for 72 hr at 37°C or seeded in 100 mm tissue-culture treated plates, grown for 24 hr at 37°C, treated with 5 nM FK866 (Sigma Aldrich), and grown for an additional 48 hr at 37°C. Total cellular RNA was isolated with TRIzol Reagent according to the manufacture’s protocol (Thermo Fisher Scientific).

### Detection and quantitation of NAD^+^- and NADH-capped mitochondrial RNA in vivo: DNAzyme cleavage

For analysis of NCIN capping of *S. cerevisiae* mitochondrial RNA, 40 µg of total cellular RNA was mixed with 1 µM DNAzyme (JB557 for *S. cerevisiae* COX2 RNA; JB526 for *S. cerevisiae* 21S RNA) in buffer containing 10 mM Tris pH 8.0, 50 mM NaCl, 2 mM DTT, and 10 mM MgCl_2_ (total volume 50 µl). When present, NudC was added to 400 nM. Reactions were incubated for 60 min at 37°C and 100 µl of stop solution and 500 µl ethanol was added. Samples were centrifuged (30 min, 21,000 g, 4°C), the supernatant removed, and the pellet resuspended in RNA loading dye.

For analysis of NCIN capping of human mitochondrial RNA, 40 µg of total cellular RNA was mixed with 1 µM DNAzyme (JB559 for human LSP-generated RNA) in buffer containing 10 mM Tris pH 8.0, 50 mM NaCl, 2 mM DTT (total volume 50 µl). Samples were heated to 85°C for 5 min, cooled to 37°C. MgCl_2_ was added to a final concentration of 10 mM and, when present, NudC was added to 400 nM. Reactions were incubated for 60 min at 37°C and 100 µl of stop solution and 500 µl ethanol was added. Samples were centrifuged (30 min, 21,000 g, 4°C), the supernatant removed, and the pellet resuspended in RNA loading dye.

To prepare synthetic RNA standards non-radiolabeled full-length RNA products were generated by in vitro transcription reactions containing 1 µM of template DNA, 1 µM of mtRNAP, 1 µM transcription factor(s), 1 mM initiating nucleotide (ATP, NAD^+^ or NADH), 200 µM GTP, 200 µM UTP, 200 µM CTP, and 20 µM ATP. Reactions were incubated for 60 min at 25°C, stopped by addition of stop solution, extracted with acid phenol:chloroform (5:1, pH 4.5; Thermo Fisher Scientific) and ethanol precipitated. Full-length RNAs were resuspended in buffer containing 10 mM Tris pH 8.0, 50 mM NaCl, 2 mM DTT, and 10 mM MgCl_2_ and treated with DNAzyme as described above.

### Detection and quantitation of NAD^+^- and NADH-capped mitochondrial RNA in vivo: hybridization with a radiolabeled oligodeoxyribonucleotide probe

NCIN capping of DNAzyme-generated subfragments of mitochondrial RNA were analyzed by a procedure consisting of: (i) electrophoresis on 7.5 M urea, 1x TBE, 10% polyacrylamide gels supplemented with 0.2% 3-acrylamidophenylboronic acid (Boron Molecular); (ii) transfer of nucleic acids to a Nytran supercharge nylon membrane (GE Healthcare Life Sciences) using a semidry transfer apparatus (Bio-Rad); (iii) immobilization of nucleic acids by UV crosslinking; (iv) incubation with a ^32^P-labelled oligodeoxyribonucleotide probe complementary to the 5'-end containing subfragments of target RNAs (JB555, COX2 RNA; JB525, 21S RNA; JB515, LSP-derived RNA; ^32^P-labelled using T4 polynucleotide kinase and [γ^32^P]-ATP [Perkin Elmer]); (v) high-stringency washing, procedures as in (Goldman et al., 2015); and (vi) storage-phosphor imaging (Typhoon 9400 variable-mode imager; GE Life Science).

Bands corresponding to uncapped and NCIN-capped DNAzyme-generated subfragments were quantified using ImageQuant software. The percentages of uncapped RNA (5'-ppp), NAD^+^-capped RNA (5'-NAD^+^), or NADH-capped RNA (5'-NADH) to total RNA were determined from three biological replicates.

### Determination of NAD(H) levels in human HEK293T cells

Cells were lysed in 400 μl of NAD/H Extraction Buffer (NAD/H Quantitation Kit, Sigma-Aldrich) and total proteins were extracted with two freeze-thaw cycles (20 min on dry ice, 10 min at RT). Lysates were centrifuged (10 min, 13,000 g, 4°C), the supernatant was isolated, and the protein concentration was determined. NAD(H) was measured using a NAD/H Quantitation Kit (Sigma) from a volume of supernatant containing 10 μg protein.

Molecules of NAD(H) per cell were calculated using a value of 300 pg total protein per cell. The cellular concentration of NAD(H) was then estimated by using a cellular volume of ~1200 µm^3^ for HEK293T cells and assuming a homogenous distribution of NAD(H) in the cell. The volume of HEK293T cells was calculated using a value of 13 µm for the diameter of HEK293T cells, assuming trypsinized cells adopt a spherical shape. NAD/H concentrations were determined from three biological replicates.

## Additional files

Transparent reporting form

### Data availability

All data generated or analysed during this study are included in the manuscript.
