# Supplementary figures and images for: Highly efficient 5' capping of mitochondrial RNA with NAD+ and NADH by yeast and human mitochondrial RNA polymerase
Source: eLife. 2018 Dec 12;7:e42179. doi: 10.7554/eLife.42179 (PMC6298784; doi:10.7554/eLife.42179)

Figure 1 – Source data

C – *Saccharomyces cerevisiae* mtRNAP

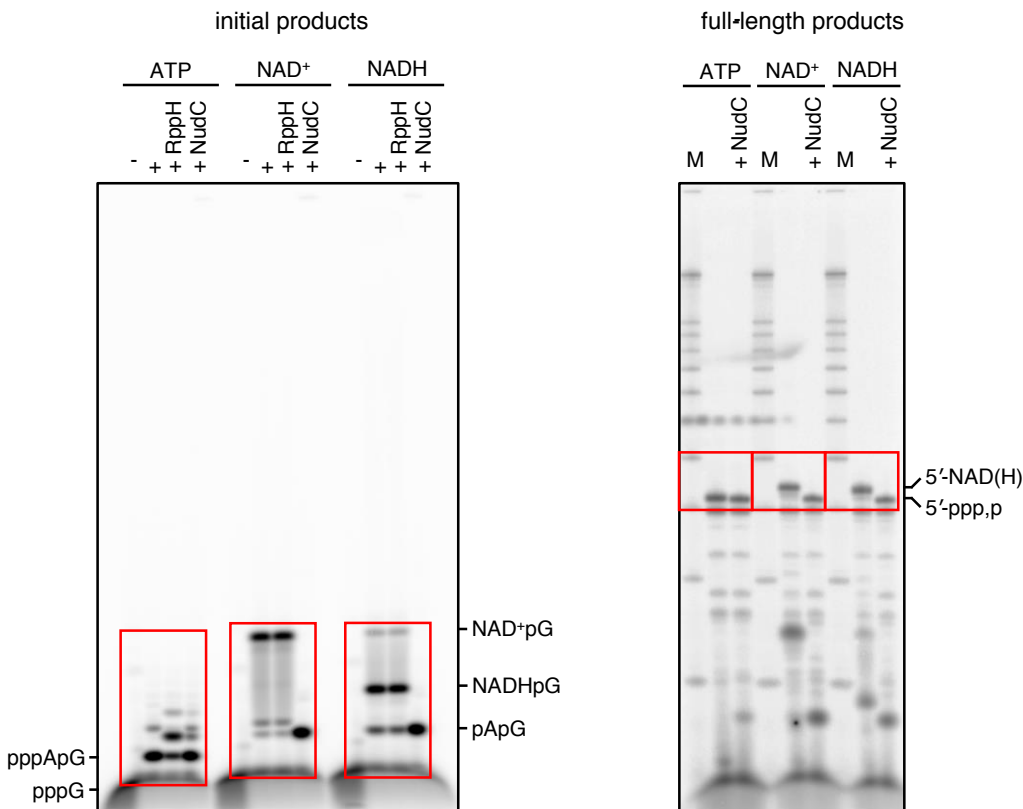

D – human mtRNAP

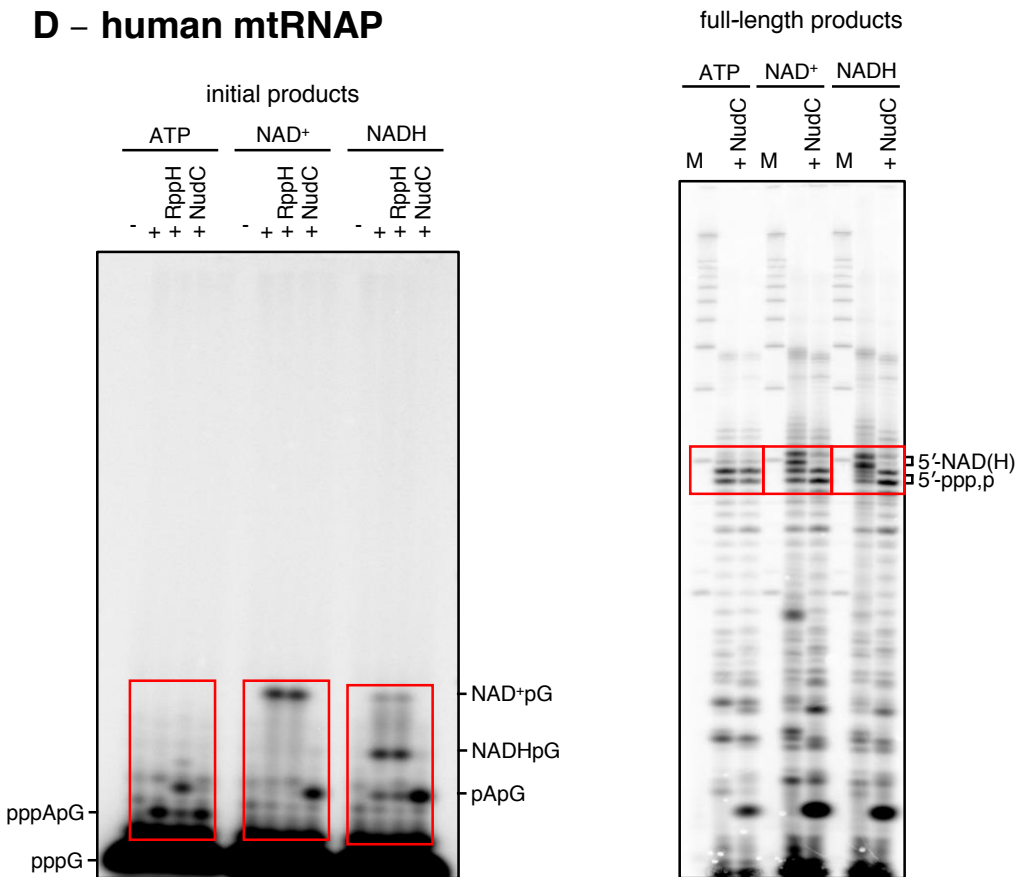

Supplement: Figure 1—source data 1. [file elife-42179-fig1-data1.pdf]

Figure 1-supplement 1 source data

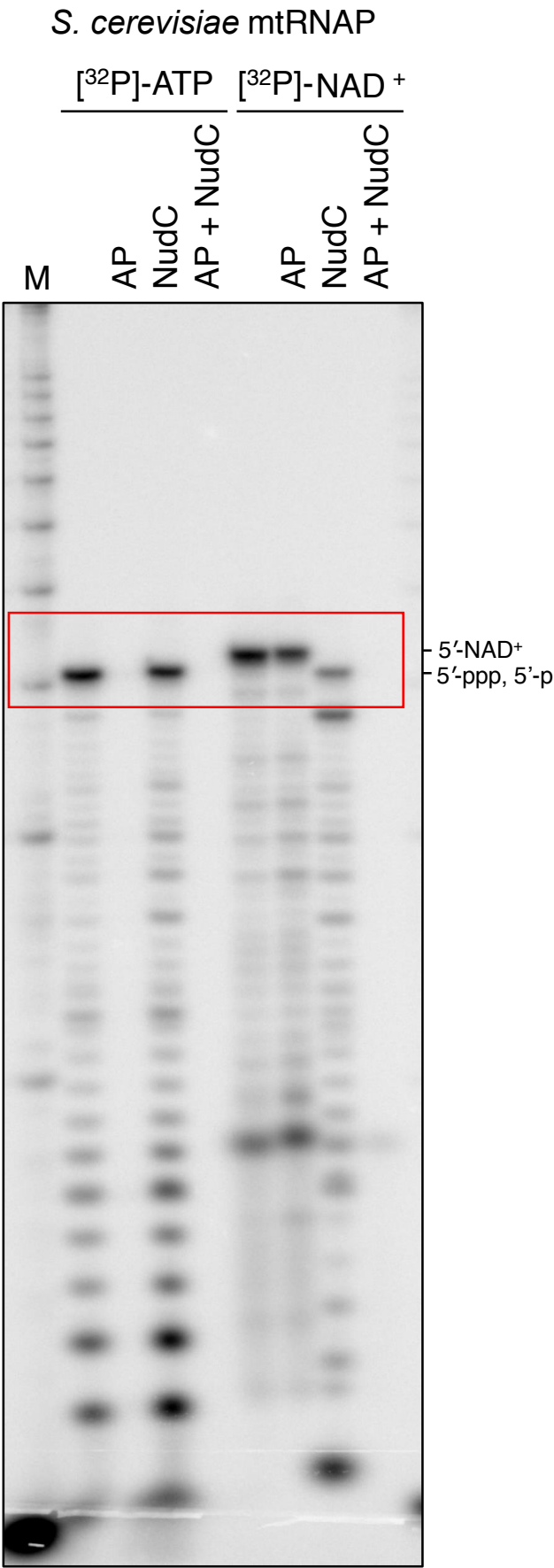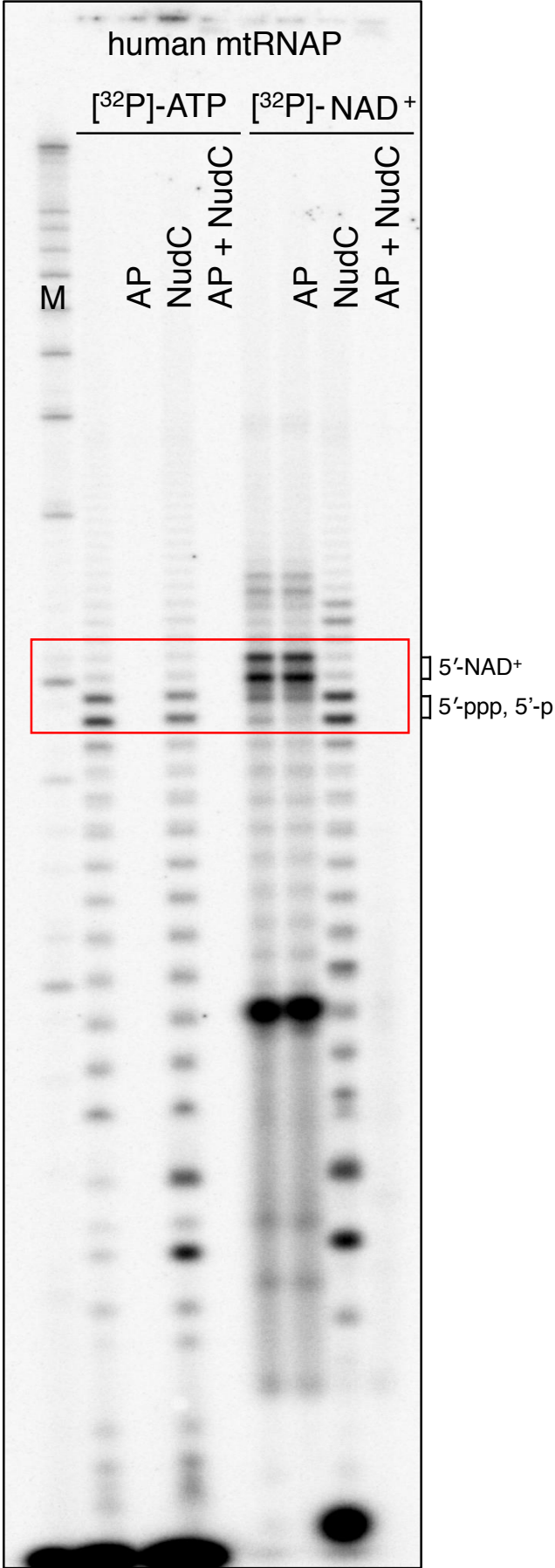

Supplement: Figure 1—source data 2. [file elife-42179-fig1-data2.pdf]

**Figure 3 – Source data**

**B – Initial product assays on +1 variant templates**

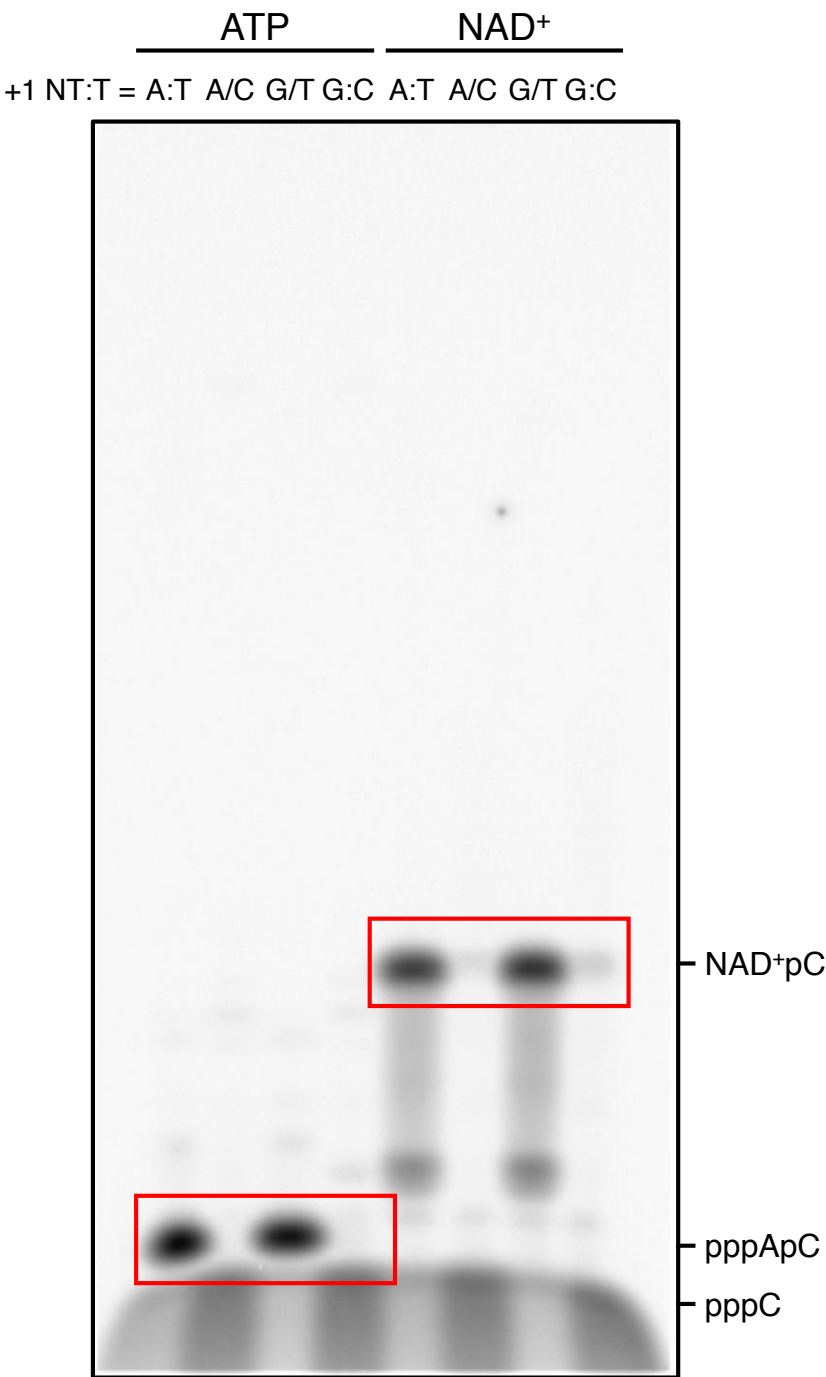

Supplement: Figure 3—source data 1. [file elife-42179-fig3-data1.pdf]

Figure 4 – Source data

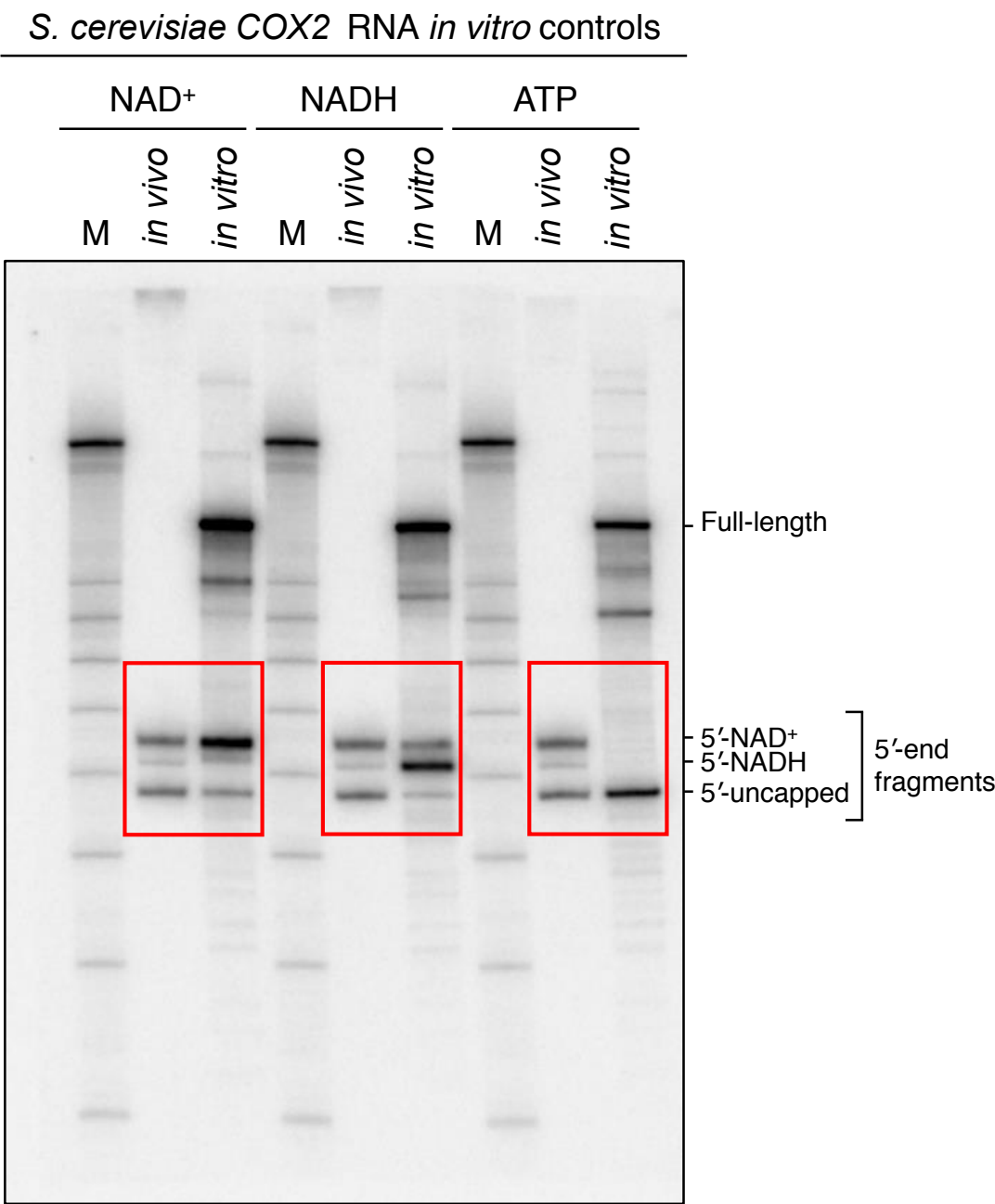

Supplement: Figure 4—source data 1. [file elife-42179-fig4-data1.pdf]

## Figure 5 – Source data

### A *S. cerevisiae*

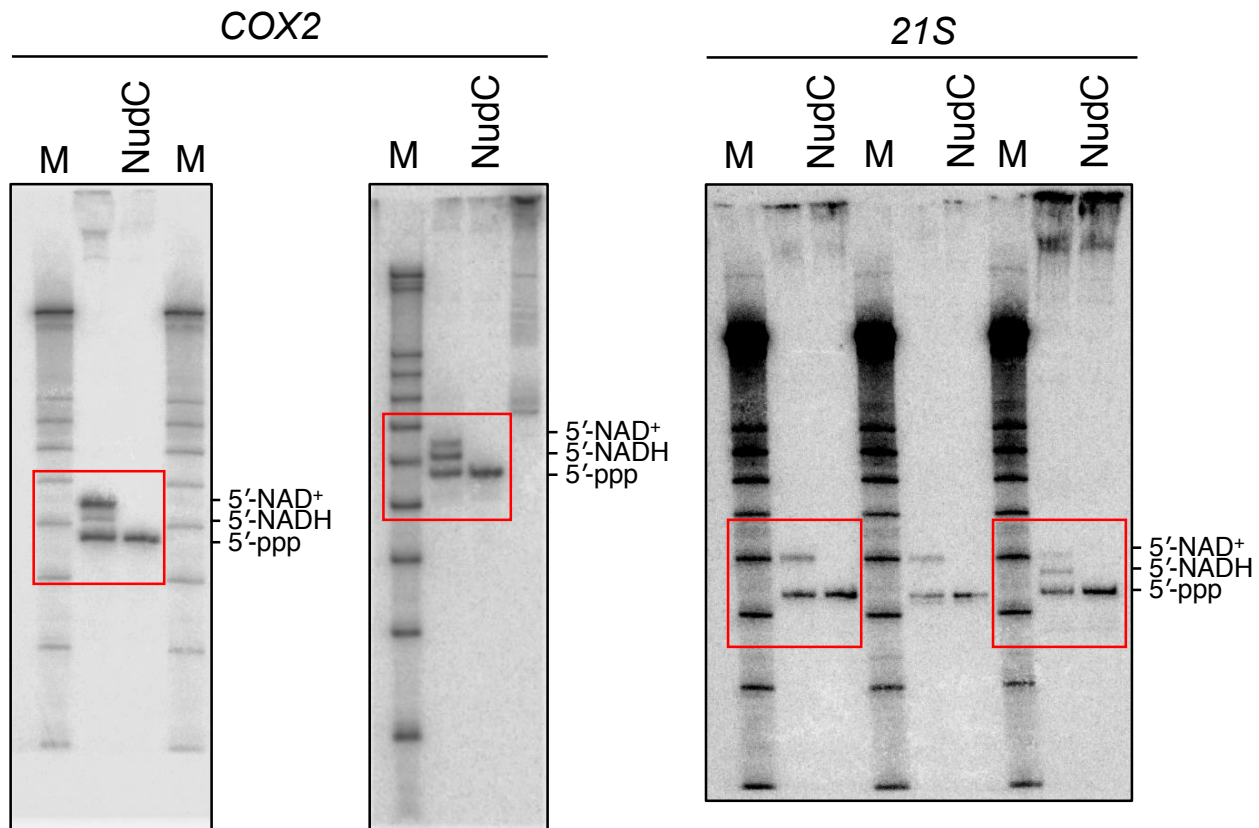

### B human (HEK293T cells)

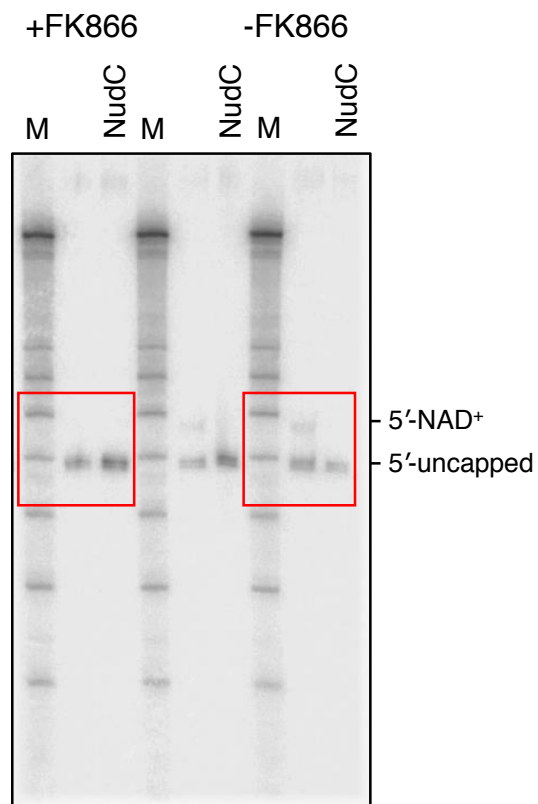

Supplement: Figure 5—source data 1. [file elife-42179-fig5-data1.pdf]
