# Supplementary material for: Highly efficient 5' capping of mitochondrial RNA with NAD+ and NADH by yeast and human mitochondrial RNA polymerase
Source: eLife. 2018 Dec 12;7:e42179. doi: 10.7554/eLife.42179 (PMC6298784; doi:10.7554/eLife.42179)
Supplement: Figure 2—source data 1. [file elife-42179-fig2-data1.pdf]

**Figure 2A**		21S										15S									
		NAD+					NADH					NAD+					NADH				
[NCIN] (μM)	[NCIN]/[ATP]	Set 1	Set 2	Set 3	Avg	SD	Set 1	Set 2	Set 3	Avg	SD	Set 1	Set 2	Set 3	Avg	SD	Set 1	Set 2	Set 3	Avg	SD
50	0.25	0.086	0.116	0.081	0.094	0.016	0.085	0.096	0.080	0.087	0.008	0.051	0.047	0.049	0.049	0.002	0.036	0.050	0.006	0.031	0.022
100	0.5	0.170	0.147	0.152	0.156	0.010	0.170	0.160	0.147	0.159	0.011	0.130	0.108	0.108	0.115	0.013	0.074	0.083	0.056	0.071	0.014
200	1	0.271	0.270	0.268	0.270	0.001	0.303	0.271	0.266	0.280	0.020	0.288	0.278	0.238	0.268	0.026	0.161	0.205	0.150	0.172	0.029
400	2	0.415	0.391	0.410	0.406	0.011	0.559	0.446	0.448	0.484	0.065	0.481	0.480	0.430	0.464	0.029	0.358	0.339	0.305	0.334	0.027
800	4	0.575	0.571	0.575	0.574	0.002	0.536	0.625	0.629	0.597	0.053	0.679	0.676	0.675	0.677	0.002	0.600	0.526	0.568	0.565	0.037
1600	8	0.702	0.704	0.696	0.701	0.003	0.768	0.773	0.763	0.768	0.005	0.819	0.806	0.809	0.811	0.007	0.731	0.704	0.711	0.715	0.014
3200	16	0.777	0.790	0.785	0.784	0.005	0.882	0.873	0.878	0.878	0.005	0.887	0.882	0.881	0.883	0.004	0.853	0.804	0.837	0.831	0.025
6400	32	0.804	0.807	0.805	0.806	0.002	0.938	0.926	0.934	0.933	0.006						0.925	0.892	0.924	0.914	0.019

**Figure 2B**		LSP										HSP1									
		NAD+					NADH					NAD+					NADH				
[NCIN] (μM)	[NCIN]/[ATP]	Set 1	Set 2	Set 3	Avg	SD	Set 1	Set 2	Set 3	Avg	SD	Set 1	Set 2	Set 3	Avg	SD	Set 1	Set 2	Set 3	Avg	SD
50	0.25	0.045	0.066	0.074	0.062	0.015	0.035	0.033	0.035	0.034	0.001	0.043	0.028	0.039	0.037	0.008	0.093	0.152	0.074	0.106	0.041
100	0.5	0.117	0.131	0.132	0.127	0.009	0.090	0.095	0.090	0.092	0.003	0.077	0.071	0.072	0.073	0.003	0.166	0.183	0.195	0.182	0.014
200	1	0.216	0.278	0.245	0.246	0.031	0.235	0.225	0.226	0.229	0.006	0.154	0.139	0.157	0.150	0.009	0.313	0.341	0.367	0.341	0.027
400	2	0.361	0.357	0.394	0.371	0.021	0.436	0.439	0.436	0.437	0.002	0.267	0.261	0.261	0.263	0.003	0.500	0.544	0.543	0.529	0.025
800	4	0.524	0.541	0.548	0.538	0.012	0.649	0.644	0.630	0.641	0.010	0.436	0.399	0.394	0.409	0.023	0.726	0.767	0.741	0.745	0.021
1600	8	0.658	0.616	0.698	0.657	0.041	0.782	0.780	0.774	0.779	0.004	0.562	0.521	0.545	0.543	0.021	0.795	0.856	0.850	0.834	0.034
3200	16						0.844	0.841	0.831	0.839	0.007						0.874	0.895	0.906	0.891	0.017
6400	32						0.844	0.837	0.838	0.840	0.004						0.879	0.919	0.939	0.912	0.030
