# Supplementary material for: Highly efficient 5' capping of mitochondrial RNA with NAD+ and NADH by yeast and human mitochondrial RNA polymerase
Source: eLife. 2018 Dec 12;7:e42179. doi: 10.7554/eLife.42179 (PMC6298784; doi:10.7554/eLife.42179)
Supplement: Figure 2—source data 2. [file elife-42179-fig2-data2.pdf]

**Figure 2C**		Sce mtRNAP				
[ATP] (μM)	[NAD+]/[ATP]	Set 1	Set 2	Set 3	Avg	SD
25	40	0.876	0.947	0.963	0.929	0.046
50	20	0.828	0.824	0.859	0.837	0.019
100	10	0.762	0.767	0.782	0.770	0.010
200	5	0.657	0.650	0.673	0.660	0.012
400	2.5	0.468	0.469	0.507	0.481	0.022
800	1.25	0.299	0.316	0.345	0.320	0.023
1600	0.625	0.181	0.176	0.194	0.184	0.009
		human mtRNAP				
[ATP] (μM)	[NAD+]/[ATP]	Set 1	Set 2	Set 3	Avg	SD
25	40	0.922	0.825	0.909	0.886	0.053
50	20	0.860	0.757	0.860	0.826	0.060
100	10	0.657	0.650	0.609	0.639	0.025
200	5	0.531	0.572	0.503	0.535	0.035
400	2.5	0.353	0.364	0.349	0.355	0.008
800	1.25	0.159	0.187	0.162	0.169	0.016
1600	0.625	0.087	0.124	0.094	0.102	0.020
		Eco RNAP				
[ATP] (μM)	[NAD+]/[ATP]	Set 1	Set 2	Set 3	Avg	SD
3.125	320	0.799	0.716	0.734	0.750	0.044
6.25	160	0.642	0.623	0.646	0.637	0.012
12.5	80	0.476	0.448	0.451	0.458	0.015
25	40	0.317	0.339	0.304	0.320	0.017
50	20	0.198	0.189	0.174	0.187	0.012
100	10	0.099	0.086	0.086	0.090	0.008
200	5	0.044	0.050	0.042	0.045	0.004
		Sce RNAP II				
[ATP] (μM)	[NAD+]/[ATP]	Set 1	Set 2	Set 3	Avg	SD
12.5	320	0.643	0.690	0.744	0.692	0.051
25	160	0.414	0.562	0.589	0.522	0.094
50	80	0.383	0.527	0.554	0.488	0.092
100	40	0.381	0.350	0.295	0.342	0.043
200	20	0.260	0.143	0.171	0.191	0.061
400	10	0.107	0.095	0.104	0.102	0.006
800	5	0.043	0.051	0.059	0.051	0.008

**Figure 2D**		Sce mtRNAP				
[ATP] (μM)	[NADH]/[ATP]	Set 1	Set 2	Set 3	Avg	SD
25	40	0.957	0.959	0.959	0.958	0.002
50	20	0.859	0.870	0.873	0.867	0.007
100	10	0.772	0.784	0.813	0.790	0.021
200	5	0.644	0.710	0.717	0.690	0.040
400	2.5	0.556	0.599	0.594	0.583	0.023
800	1.25	0.446	0.432	0.500	0.459	0.036
1600	0.625	0.306	0.297	0.327	0.310	0.016
		human mtRNAP				
[ATP] (μM)	[NADH]/[ATP]	Set 1	Set 2	Set 3	Avg	SD
25	40	0.983	0.967	0.977	0.976	0.008
50	20	0.929	0.958	0.939	0.942	0.015
100	10	0.871	0.911	0.847	0.876	0.032
200	5	0.769	0.731	0.716	0.739	0.027
400	2.5	0.590	0.580	0.517	0.562	0.040
800	1.25	0.387	0.367	0.332	0.362	0.028
1600	0.625	0.227	0.231	0.180	0.213	0.028
		Eco RNAP				
[ATP] (μM)	[NADH]/[ATP]	Set 1	Set 2	Set 3	Avg	SD
3.125	320	0.937	0.922	0.950	0.936	0.014
6.25	160	0.858	0.831	0.858	0.849	0.016
12.5	80	0.728	0.752	0.756	0.745	0.015
25	40	0.565	0.551	0.588	0.568	0.019
50	20	0.386	0.383	0.387	0.385	0.002
100	10	0.214	0.209	0.225	0.216	0.009
200	5	0.123	0.112	0.114	0.116	0.006
		Sce RNAP II				
[ATP] (μM)	[NADH]/[ATP]	Set 1	Set 2	Set 3	Avg	SD
12.5	320	0.949	0.961	0.927	0.946	0.017
25	160	0.965	0.923	0.904	0.931	0.031
50	80	0.948	0.834	0.819	0.867	0.071
100	40	0.687	0.635	0.702	0.675	0.035
200	20	0.487	0.483	0.519	0.497	0.019
400	10	0.218	0.242	0.231	0.231	0.012
800	5	0.127	0.123	0.126	0.126	0.002

**Figure 2-supplement 2**		T7 RNAP				
[ATP] (μM)	[NAD+]/[ATP]	Set 1	Set 2	Set 3	Avg	SD
25	40	0.905	0.911	0.921	0.912	0.008
50	20	0.813	0.817	0.835	0.822	0.012
100	10	0.663	0.661	0.675	0.667	0.008
200	5	0.479	0.497	0.511	0.496	0.016
400	2.5	0.280	0.300	0.310	0.296	0.015
800	1.25	0.159	0.164	0.161	0.162	0.003
1600	0.625	0.077	0.081	0.074	0.077	0.003
[ATP] (μM)	[NADH]/[ATP]	Set 1	Set 2	Set 3	Avg	SD
25	40	0.977	0.972	0.973	0.974	0.002
50	20	0.937	0.930	0.933	0.933	0.004
100	10	0.851	0.832	0.845	0.843	0.010
200	5	0.702	0.685	0.729	0.705	0.022
400	2.5	0.529	0.502	0.518	0.516	0.013
800	1.25	0.344	0.329	0.357	0.343	0.014
1600	0.625	0.170	0.197	0.201	0.189	0.017
