# Supplementary material for: Highly efficient 5' capping of mitochondrial RNA with NAD+ and NADH by yeast and human mitochondrial RNA polymerase
Source: eLife. 2018 Dec 12;7:e42179. doi: 10.7554/eLife.42179 (PMC6298784; doi:10.7554/eLife.42179)
Supplement: Figure 3—source data 2. [file elife-42179-fig3-data2.pdf]

Figure 3C		-1A:T					-1T:A					-1G:C					-1C:G				
[ATP] (mM)	[NAD+]/[ATP]	Set 1	Set 2	Set 3	Avg	SD	Set 1	Set 2	Set 3	Avg	SD	Set 1	Set 2	Set 3	Avg	SD	Set 1	Set 2	Set 3	Avg	SD
25	40	0.962	0.947	0.957	0.955	0.008	0.935	0.942	0.941	0.939	0.004	0.948	0.930	0.931	0.936	0.010	0.873	0.896	0.895	0.888	0.013
50	20	0.906	0.891	0.894	0.897	0.008	0.867	0.821	0.829	0.839	0.025	0.889	0.870	0.843	0.867	0.023	0.742	0.786	0.752	0.760	0.023
100	10	0.805	0.734	0.758	0.766	0.036	0.673	0.632	0.654	0.653	0.020	0.791	0.683	0.697	0.724	0.059	0.562	0.617	0.552	0.577	0.035
200	5	0.691	0.575	0.567	0.611	0.069	0.396	0.448	0.450	0.431	0.031	0.656	0.543	0.544	0.581	0.065	0.435	0.440	0.352	0.409	0.049
400	2.5	0.472	0.410	0.379	0.420	0.048	0.167	0.224	0.253	0.215	0.044	0.465	0.331	0.335	0.377	0.076	0.232	0.259	0.206	0.233	0.027
800	1.25	0.342	0.223	0.202	0.256	0.075	0.140	0.126	0.133	0.133	0.007	0.309	0.242	0.219	0.256	0.047	0.133	0.158	0.113	0.134	0.023
1600	0.625	0.195	0.115	0.124	0.145	0.044	0.096	0.061	0.071	0.076	0.018	0.232	0.135	0.106	0.158	0.066	0.073	0.133	0.056	0.087	0.040

Figure 3D		-1*/T					-1*/A					-1*/C					-1*/G				
[ATP] (μM)	[NAD+]/[ATP]	Set 1	Set 2	Set 3	Avg	SD	Set 1	Set 2	Set 3	Avg	SD	Set 1	Set 2	Set 3	Avg	SD	Set 1	Set 2	Set 3	Avg	SD
25	40	0.928	0.958	0.945	0.944	0.015	0.909	0.938	0.913	0.920	0.015	0.914	0.951	0.964	0.943	0.026	0.857	0.899	0.869	0.875	0.022
50	20	0.889	0.909	0.895	0.898	0.010	0.828	0.679	0.843	0.783	0.090	0.864	0.899	0.919	0.894	0.028	0.744	0.837	0.746	0.776	0.053
100	10	0.766	0.806	0.810	0.794	0.025	0.649	0.667	0.687	0.668	0.019	0.727	0.855	0.813	0.799	0.065	0.554	0.592	0.584	0.577	0.020
200	5	0.637	0.639	0.664	0.647	0.015	0.476	0.473	0.508	0.486	0.019	0.660	0.761	0.682	0.701	0.053	0.406	0.374	0.424	0.401	0.025
400	2.5	0.476	0.436	0.504	0.472	0.034	0.301	0.358	0.348	0.336	0.030	0.452	0.545	0.492	0.496	0.047	0.232	0.233	0.234	0.233	0.001
800	1.25	0.251	0.295	0.303	0.283	0.028	0.153	0.189	0.187	0.176	0.020	0.249	0.288	0.291	0.276	0.024	0.122	0.182	0.141	0.148	0.030
1600	0.625	0.164	0.213	0.184	0.187	0.025	0.087	0.117	0.110	0.105	0.016	0.162	0.208	0.192	0.188	0.023	0.078	0.061	0.080	0.073	0.011

Figure 3-supplement 1		-1A:T					-1T:A					-1G:C					-1C:G				
[ATP] (μM)	[NAD+]/[ATP]	Set 1	Set 2	Set 3	Avg	SD	Set 1	Set 2	Set 3	Avg	SD	Set 1	Set 2	Set 3	Avg	SD	Set 1	Set 2	Set 3	Avg	SD
25	40	0.903	0.922	0.902	0.909	0.011	0.869	0.890	0.852	0.870	0.019	0.897	0.932	0.924	0.917	0.018	0.852	0.866	0.860	0.859	0.007
50	20	0.810	0.822	0.814	0.815	0.006	0.767	0.797	0.751	0.772	0.023	0.849	0.868	0.859	0.859	0.009	0.713	0.772	0.697	0.727	0.039
100	10	0.734	0.751	0.691	0.725	0.031	0.620	0.642	0.613	0.625	0.015	0.729	0.766	0.746	0.747	0.018	0.608	0.609	0.524	0.580	0.049
200	5	0.646	0.642	0.619	0.636	0.015	0.503	0.531	0.429	0.488	0.053	0.603	0.635	0.624	0.621	0.016	0.492	0.531	0.441	0.488	0.045
400	2.5	0.460	0.445	0.420	0.442	0.020	0.314	0.336	0.311	0.320	0.013	0.419	0.446	0.484	0.450	0.033	0.337	0.335	0.282	0.318	0.032
800	1.25	0.314	0.250	0.238	0.267	0.041	0.178	0.183	0.161	0.174	0.012	0.268	0.281	0.284	0.278	0.009	0.241	0.160	0.138	0.180	0.054
1600	0.625	0.270	0.154	0.157	0.194	0.066	0.116	0.073	0.106	0.099	0.022	0.169	0.147	0.178	0.165	0.016	0.165	0.094	0.076	0.112	0.047

Figure 3E	mtRNAP		*E. coli* RNAP		T7 RNAP	
-1	Avg	SD	Avg	SD	Avg	SD
A:T	0.300	0.009	0.095	0.005	0.323	0.029
G:C	0.277	0.006	0.106	0.003	0.319	0.013
T:A	0.175	0.004	0.046	0.001	0.180	0.010
C:G	0.134	0.003	0.014	0.001	0.173	0.019
