# Supplementary material for: Highly efficient 5' capping of mitochondrial RNA with NAD+ and NADH by yeast and human mitochondrial RNA polymerase
Source: eLife. 2018 Dec 12;7:e42179. doi: 10.7554/eLife.42179 (PMC6298784; doi:10.7554/eLife.42179)
Supplement: Figure 5—source data 2. [file elife-42179-fig5-data2.pdf]

COX2 (aerobic respiration)				COX2 (anaerobic fermentation)			
Replicate	%ppp	%NAD	%NADH	Replicate	%ppp	%NAD	%NADH
1	44.5	48.0	0.1	1	32.6	27.1	40.3
2	48.9	42.9	0.1	2	32.9	23.2	43.9
3	39.4	52.5	0.1	3	37.3	18.9	43.8
Avg	44.2	47.8	0.1	Avg	34.3	23.1	42.7
SD	4.7	4.8	0.0	SD	2.6	4.1	2.1

21S (aerobic respiration)				21S (anaerobic fermentation)			
Replicate	%ppp	%NAD	%NADH	Replicate	%ppp	%NAD	%NADH
1	78.3	21.7	n/a	1	59.9	15.2	24.9
2	65.8	34.2	n/a	2	71.6	8.3	20.1
3	71.8	28.2	n/a	3	68.6	12.1	19.2
Avg	72.0	28.0		Avg	66.7	11.9	21.4
SD	6.2	6.2		SD	6.1	3.4	3.1

7S RNA (HEK293T cells)			
Replicate	%ppp	%NAD	%NADH
1	80.2	19.8	n/a
2	84.0	16.0	n/a
3	83.3	16.7	n/a
Avg	82.5	17.5	
SD	2.0	2.0	
